# Identification of the Bacterial Biosynthetic Gene Clusters of the Oral Microbiome Illuminates the Unexplored Social Language of Bacteria during Health and Disease

**DOI:** 10.1128/mBio.00321-19

**Published:** 2019-04-16

**Authors:** Gajender Aleti, Jonathon L. Baker, Xiaoyu Tang, Ruth Alvarez, Márcia Dinis, Nini C. Tran, Alexey V. Melnik, Cuncong Zhong, Madeleine Ernst, Pieter C. Dorrestein, Anna Edlund

**Affiliations:** a Genomic Medicine Group, J. Craig Venter Institute, La Jolla, California, USA; b School of Dentistry, The University of California, Los Angeles, California, USA; c Department of Electric Engineering and Computer Science, The University of Kansas, Lawrence, Kansas, USA; d Collaborative Mass Spectrometry Innovation Center, Skaggs School of Pharmacy and Pharmaceutical Sciences, The University of California San Diego, La Jolla, California, USA; Academic Center for Dentistry Amsterdam; Fred Hutchinson Cancer Research Center

**Keywords:** biosynthetic gene clusters, caries, genome mining, oral microbiome, periodontitis, small molecules

## Abstract

Small molecules are the primary communication media of the microbial world. Recent bioinformatic studies, exploring the biosynthetic gene clusters (BGCs) which produce many small molecules, have highlighted the incredible biochemical potential of the signaling molecules encoded by the human microbiome. Thus far, most research efforts have focused on understanding the social language of the gut microbiome, leaving crucial signaling molecules produced by oral bacteria and their connection to health versus disease in need of investigation. In this study, a total of 4,915 BGCs were identified across 461 genomes representing a broad taxonomic diversity of oral bacteria. Sequence similarity networking provided a putative product class for more than 100 unclassified novel BGCs. The newly identified BGCs were cross-referenced against 254 metagenomes and metatranscriptomes derived from individuals either with good oral health or with dental caries or periodontitis. This analysis revealed 2,473 BGCs, which were differentially represented across the oral microbiomes associated with health versus disease. Coabundance network analysis identified numerous inverse correlations between BGCs and specific oral taxa. These correlations were present in healthy individuals but greatly reduced in individuals with dental caries, which may suggest a defect in colonization resistance. Finally, corroborating mass spectrometry identified several compounds with homology to products of the predicted BGC classes. Together, these findings greatly expand the number of known biosynthetic pathways present in the oral microbiome and provide an atlas for experimental characterization of these abundant, yet poorly understood, molecules and socio-chemical relationships, which impact the development of caries and periodontitis, two of the world’s most common chronic diseases.

## Abstract (precis)

The healthy oral microbiome is symbiotic with the human host, importantly providing colonization resistance against potential pathogens. Dental caries and periodontitis are two of the world’s most common and costly chronic infectious diseases and are caused by a localized dysbiosis of the oral microbiome. Bacterially produced small molecules, often encoded by BGCs, are the primary communication media of bacterial communities and play a crucial, yet largely unknown, role in the transition from health to dysbiosis. This study provides a comprehensive mapping of the BGC repertoire of the human oral microbiome and identifies major differences in health compared to disease. Furthermore, BGC representation and expression is linked to the abundance of particular oral bacterial taxa in health versus dental caries and periodontitis. Overall, this study provides a significant insight into the chemical communication network of the healthy oral microbiome and how it devolves in the case of two prominent diseases.

## INTRODUCTION

The human body is inhabited by rich and diverse bacterial communities, which are intimately linked to the health of the human host (1). Small molecules, which are often encoded by biosynthetic gene clusters (BGCs), are the primary means of communication in this microbial world. Recent studies suggest that the human microbiota has the potential to synthesize structurally diverse small molecules and that these small molecules serve as mediators in a variety of microbe-microbe and host-microbe interactions (2–4). These interactions include antibacterial activity (5), bacterial signaling (6), immune modulation (7), biofilm formation (8, 9), host colonization (10), nutrient scavenging (11), and stress protection (12). Disruption of the finely tuned equilibrium of the bacterial ecosystems in the human microbiome, referred to as dysbiosis, is associated with a plethora of diseases. While the mechanistic underpinnings of a shift to a dysbiotic community remain poorly understood, there is little doubt that signaling via the small molecules produced by microbial BGCs plays a critical role in the transition to dysbiosis and associated pathogenesis (13, 14).

The human oral cavity contains an assortment of ecological niches, and as such, harbors one of the most diverse microbial populations in the human body (1, 15). Dental caries and periodontitis are two of the most common and costly chronic conditions afflicting humans and are the result of localized dysbiosis in the oral cavity (16–20). Unlike the rest of the human digestive tract, the oral cavity is consistently exposed to the exterior environment. Therefore, an indispensable portion of the first line of defense against invading pathogens is the colonization resistance provided by a healthy oral microbiome. Indeed, dysbiosis of the oral microbiome is not only directly linked to oral diseases but is also implicated in system-wide health (21), stressing the urgent need to unravel the underlying factors that shape and maintain a healthy human oral microbiome.

Elucidating the transmissions relayed by oral bacterial small molecules could lead to a deeper understanding of key ecological factors that set the stage for oral community succession, in health and pathogenesis. A large and growing body of literature suggests that the microbial composition and metabolic potential of the saliva and dental plaque varies significantly in healthy versus disease states (22–28). Therefore, we hypothesize that the abundance and expression of BGCs, which produce small molecules, may drive crucial bacterial interactions which contribute to health or disease. To explore this further, the biosynthetic capacity of 461 well-annotated oral bacterial genomes was investigated, and an enormous diversity of BGCs was revealed. In addition, sequence reads from 294 publicly available metagenomes and metatranscriptomes, which were associated with health, dental caries, or periodontitis, were mapped to these novel oral BGCs. This analysis identified 2,473 biosynthetic pathways which were differentially represented in health versus disease. In addition, the BGC content in salivary metagenomes obtained from 24 healthy children and 23 children with dental caries was analyzed. A Bayesian network approach was employed to identify both positive and inverse correlations between BGCs and bacterial taxa, which revealed differentially abundant signaling networks and species in health compared to dental caries. Overall, this study provides significant insight into the chemical communication network of the healthy oral microbiome and how it devolves in the case of dental caries and periodontitis.

## RESULTS AND DISCUSSION

### The human oral microbiome encodes thousands of diverse BGCs from an array of species.

**FIG 1:**
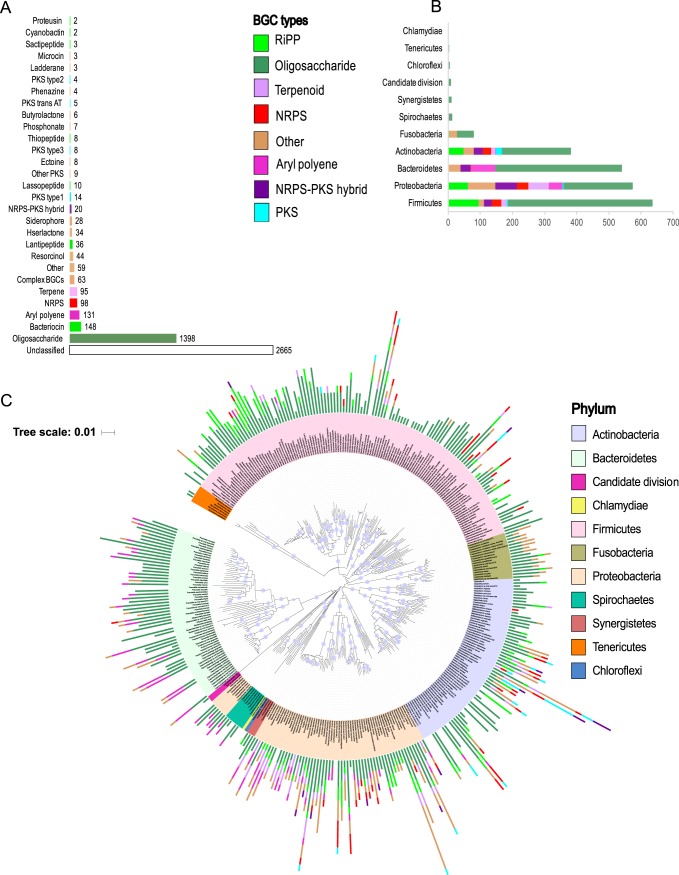
The oral microbiome contains a massive diversity of BGCs encoded by a multitude of taxa. (A) Bar graph illustrating the most common BGC subtypes identified in this study. Bars are colored according to higher level BGC class. (B) Bar graph illustrating the distribution of eight major classes of BGCs by phyla. (C) Phylogenetic tree based on 16S rRNA gene sequences showing the distribution of BGCs encoded by oral bacteria. Nodes with bootstrap values higher than 80% are displayed in the tree. The numbers of BGC types identified within each genome are shown in the bar graph and colored by BGC type. Leaf labels are colored by phyla. antiSMASH often identifies BGCs that encompass multiple gene clusters of different types fused into a single large gene cluster. Sixty-three (∼3%) of such unresolved BGCs were encountered and categorized as the “complex” BGC type (for convenience, we combined these BGCs with BGC types “Other” for subsequent analysis).

To explore the metabolic capacity of the human oral microbiome in depth, a comprehensive pipeline for mining bacterial genomes was established, utilizing antiSMASH infrastructure v4 (accessible at https://antismash.secondarymetabolites.org/) (29), including MultiGeneBlast (30). An oral bacterial genome sequence database was assembled to include a total of 461 well-curated and annotated bacterial genomes, representing 113 unique bacterial genera and 298 taxonomically unique species, as well as 72 taxa unclassified at the species level (see Table S1 posted at https://massive.ucsd.edu/ProteoSAFe/status.jsp?task=1ed182b005a74b94ac730f769b2f37b1). A few genomes representing transient exogenous bacteria (e.g., legume symbiont Mesorhizobium loti, pathogenic Yersinia pestis), which also have oral taxon IDs in the Human Oral Microbiome Database (HOMD) were included in our analysis to represent an additional source of unexplored BGC diversity. However, our sequence read mapping exercises (both with regards to metagenomic and metatranscriptomic reads) showed that most of these BGCs were of less importance, indicating that the majority were either of low abundance or absent in the oral cavity (Table S2 posted at https://massive.ucsd.edu/ProteoSAFe/status.jsp?task=1ed182b005a74b94ac730f769b2f37b1). Genomes were selected based on their completeness and level of annotation. A single genome sequence for each bacterial species was included to circumvent the overrepresentation of BGCs from bacteria with a high number of genome representatives. Indeed, in a previous bioinformatic study of 169 Streptococcus mutans genomes, ∼1,000 putative BGCs were identified, revealing an incredible potential to produce small molecules within one bacterial species (31). Therefore, it should be noted that the estimated BGC diversity reported here is likely underestimated. Clearly, strain-level diversity is important to explore in future studies. However, this will require extensive genome sequencing, since to date, most oral bacterial species lack multiple reference genomes. By applying the genome-mining pipeline described above, a total of 4,915 BGCs of known and unknown types were identified (Table S1 posted at https://massive.ucsd.edu/ProteoSAFe/status.jsp?task=1ed182b005a74b94ac730f769b2f37b1). BGCs annotated as fatty acid synthases, which are often involved in primary metabolism, were excluded. Approximately 50% of the identified BGCs were of an unknown class, congruent with the observations of other efforts to identify BGCs (Table S1 posted at the above website) (2). The remaining 50% of BGCs (2,250) shared sequence similarities with an extensive range of previously characterized BGC classes, which is likely reflective of the high taxonomic diversity observed within the oral cavity compared to many other body sites (1) (Fig. 1A).

Of the BGCs of a known class, a substantial fraction (1,398 BGCs [62%]) were annotated as oligosaccharides, making it the most abundant class of BGCs in the oral cavity. Oligosaccharide pathways are widely distributed across bacterial phyla and are predominant in *Firmicutes*, *Proteobacteria*, *Bacteroidetes*, *Actinobacteria*, and *Fusobacteria*, with the highest number being identified in *Firmicutes* (Fig. 1B and C). However, the high abundance of *Firmicutes* BGCs may reflect that *Streptococcus* genomes are highly sequenced compared to genomes of other oral taxa which may have added bias to the analysis. The ecological roles of oligosaccharides are underexplored, but studies show important functions such as capsule formation in virulence development (32) and attachment to surfaces, including neighboring bacterial species and host cells (33). Furthermore, diffusible oligosaccharides are known to display antibacterial activities (34); for example, a previous study showed that polysaccharide A from the human gut bacterium Bacteroides fragilis can modulate the gut mucosal immune response (35, 36).

Another highly represented BGC class was ribosomally synthesized and posttranslationally modified peptides (RiPPs), for which 209 BGCs (9.3% of BGCs of a known class) were identified. RiPPs include molecules such as bacteriocins, lantipeptides, sactipeptides, cyanobactins, and proteusins (denoted as fluorescent green in Fig. 1). Of these RiPP types, bacteriocin-encoding BGCs were the most abundant, as they contributed ∼75% of the total RiPP diversity. Interestingly, although bacteriocin-producing BGCs were abundant in the oral microbiome overall, they were depleted in all *Bacteroidetes* genomes (Fig. 1C). The role of RiPPs, such as the bacteriocins, demands further exploration, as they exhibit antagonistic activities against other microbes sharing the same ecological niche and influence competition for persistence between commensals and pathogens (37, 38). Furthermore, multiple studies of genetic transformation in *Streptococcus* show that competence is tightly linked to bacteriocin production (39), which suggests that these molecules also play important roles in the horizontal transfer of genes and ultimately in niche differentiation and population structure changes.

BGCs encoding aryl polyene-like molecules in several *Bacteroidetes* and *Proteobacteria* genomes were identified (131 BGCs or 5.8% of BGCs of a known class). Aryl polyenes are predicted to function as protective agents against oxidative stress (40). However, only a few candidates have been experimentally characterized, leaving this group of small molecules highly underexplored. A diversity of nonribosomal peptide synthetases (NRPSs), polyketide synthase (PKS), and NRPS-PKS hybrid BGCs (ranging between 0.9% and 4.4% of BGCs of a known class) were identified, in line with a prior study, which classified BGCs in the human microbiome in multiple body habitats (2). In the same study, it was also observed that BGCs show biogeographic signatures within the oral cavity, which suggests that different social signaling skills are required to inhabit different ecological niches. NRPS and PKS compound classes are known for their antimicrobial activities and were previously characterized as possessing various nutrient scavenging, immunosuppressant, surfactant, and cytotoxic properties (41). BGCs of the terpene class were also identified (95 BGCs, 4.2% of BGCs of a known class). This diverse group of small molecules may also be of ecological and medicinal interest, since their activities have been reported as both anti-inflammatory and antimicrobial (42). The class “other” encompasses BGCs that fall outside the known categories of antiSMASH annotation, includes rare classes found in only a few species, and constituted 9.4% of the total BGCs identified (Fig. 1A). Taken together, these results show that the oral microbiome encodes a vast and highly diverse array of small molecules that have largely unexplored, yet likely pivotal, roles in ecology and health.

### Sequence similarity networks reveals distant and close homologies to well-studied classes of BGCs.

**FIG 2:**
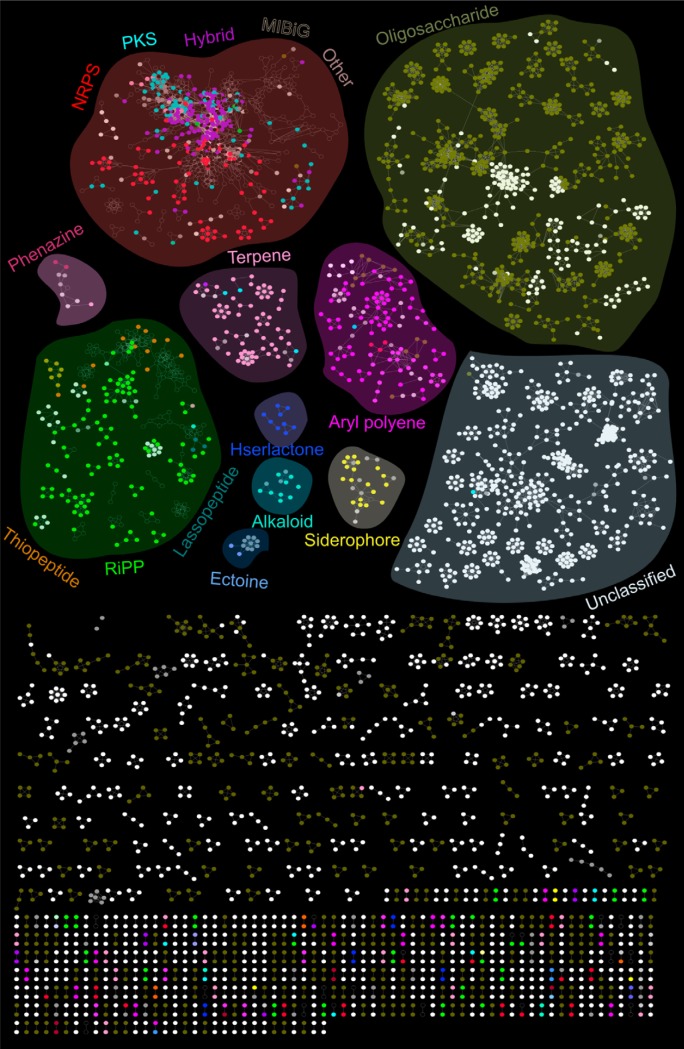
Similarity networking identified putative product classes for novel BGCs. Similarity networks between the BGCs identified in the oral cavity and the experimentally characterized reference BGCs obtained from the MIBiG repository are depicted. Subnetworks representing major BGC classes, as determined by antiSMASH and BiG-SCAPE, are highlighted with different background colors to visualize BGCs as constellations within the biosynthetic landscape. Nodes (small circles) represent amino acid sequences of BGC domains and are colored by BGC class. Unfilled nodes represent reference BGCs from the MIBiG repository. Edges drawn between the nodes correspond to pairwise distances, computed by BiG-SCAPE as the weighted combination of the Jaccard, adjacency, and domain sequence similarity indices. For increased simplicity, only subclusters of unclassified and oligosaccharide BGCs with a minimum number of eight nodes are organized into given highlighted constellation.

In order to assess the evolutionary relationships between conserved domains in the proteins encoded by BGCs, as well as to group BGCs of similar putative function to evaluate novelty, a sequence similarity network approach was applied (see Text S1 in the supplemental material). Briefly, the BGCs that were identified from the bacterial genomes using antiSMASH were aligned to the MIBiG repository (43) of 1,409 experimentally validated reference BGCs using the BiG-SCAPE algorithm (https://git.wageningenur.nl/medema-group/BiG-SCAPE). The resulting network comprised 4,242 nodes and 19,847 connecting edges, revealing both close and distant homology to characterized biosynthetic pathways (Fig. 2). Notably, a significant fraction of the previously unclassified BGCs did subnetwork with BGCs predicted to be of a known class, particularly the oligosaccharide, RiPP, and aryl polyene classes (Fig. 2). These data provide inferences as to the function of more than 100 previously unclassified novel BGCs.

10.1128/mBio.00321-19.8 TEXT S1 Supplemental results and methods. Download Text S1, DOCX file, 0.1 MB. Copyright © 2019 Aleti et al. 2019 Aleti et al. This content is distributed under the terms of the Creative Commons Attribution 4.0 International license.

The largest subnetwork, comprised of mainly oligosaccharide-encoding BGCs, showed no significant homology with any experimentally validated BGCs in the MIBiG repository (Fig. 2). This may be due to the fact that oligosaccharide-producing BGCs are at times categorized with primary metabolism, and not natural product-producing BGCs, as is the case in this study. The second-largest major subnetwork was comprised of primarily unclassified BGCs. These may encompass distinct chemical scaffolds and may represent a rich source of novel BGC types. The NRPS, PKS, NRPS-PKS hybrids, and a few terpene BGCs, grouped together, forming a subnetwork implying a set of common core domains involved in these biosynthetic assembly lines as described previously (4, 41). The majority of NRPS, PKS, NRPS-PKS hybrids, and RiPPs (in particular thiopeptides and lantipeptides) showed strong associations with MIBiG reference BGC sequences. It should be noted that these are the most prevalent classes in the MIBiG repository (Table S1 posted at https://massive.ucsd.edu/ProteoSAFe/status.jsp?task=1ed182b005a74b94ac730f769b2f37b1). Currently, only four experimentally characterized aryl polyene BGCs exist in the MIBiG database; therefore, it was not surprising that none of the nodes in the aryl polyene cluster subnetworked with MIBiG reference BGCs. Given that aryl polyenes are thought to be the most abundant BGC class in the human microbiome (4), this indicates that this class of molecules is severely understudied (Fig. 1; see also Table S1 posted at https://massive.ucsd.edu/ProteoSAFe/status.jsp?task=1ed182b005a74b94ac730f769b2f37b1). Several BGCs annotated as saccharides, other, unclassified, PKS and NRPS BGC types grouped with aryl polyene BGCs, which may represent novel hybrid classes of BGCs. Other small subnetworks include biosynthesis of terpene phenazine, homoserine lactone, alkaloid, siderophore, and ectoine. These subnetworks did not associate with MIBiG reference BGCs, indicating that they also await experimental validation. Our implemented analysis approach, using the MIBiG/BiG-SCAPE pipeline, is powerful with regard to predicting the functions of novel BGCs. The annotations we generated here provide deeper insights of which BGCs and compound classes are most likely to be identified in futures studies, due to knowledge of their closest neighbor’s biochemical properties. The BGCs remaining with completely unknown functions represent exciting future challenges, which could be addressed by generating large-insert BGC expression libraries.

While antiSMASH and network analysis were employed for broad classification of BGCs into known classes, MultiGeneBlast was also utilized at the level of the entire gene cluster to further annotate BGCs in depth and identify homologs against the MIBiG repository (30). Using this approach, the 4,915 BGCs were classified into four major categories based upon the level of homology to known experimentally validated BGCs in the MIBiG repository. This categorization resulted in 1,146 (20%) BGCs closely homologous, 848 (15%) BGCs moderately homologous, and 2,221 (40%) BGCs distantly homologous to well-characterized BGCs (see Fig. S1 in the supplemental material). A total of 1,393 (∼25%) BGCs did not appear to have significant homology to BGCs in MIBiG, based upon the E value (see Materials and Methods for details). Such a detailed annotation of BGCs harbored by the human oral microbiome has not been accomplished previously.

10.1128/mBio.00321-19.1 FIG S1 Analysis of potential chemical space encoded in BGCs with significant sequence homology (cumulative BLAST bit score of ≥1,000) to known biosynthetic pathways. The *x* axis represents the level of sequence homology, i.e., cumulative BLAST bit scores, and the *y* axis represents the known BGCs from the MIBiG repository. (A) BGCs with high homology to MIBiG BGCs (cumulative BLAST bit score was >3,000). (B) BGCs with moderate homology to MIBiG BGCs (cumulative BLAST bit score of >1,000 and <3,000). Download FIG S1, PDF file, 0.9 MB. Copyright © 2019 Aleti et al. 2019 Aleti et al. This content is distributed under the terms of the Creative Commons Attribution 4.0 International license.

### Specific BGCs and bacterial taxa are associated with periodontitis and dental caries.

**FIG 3:**
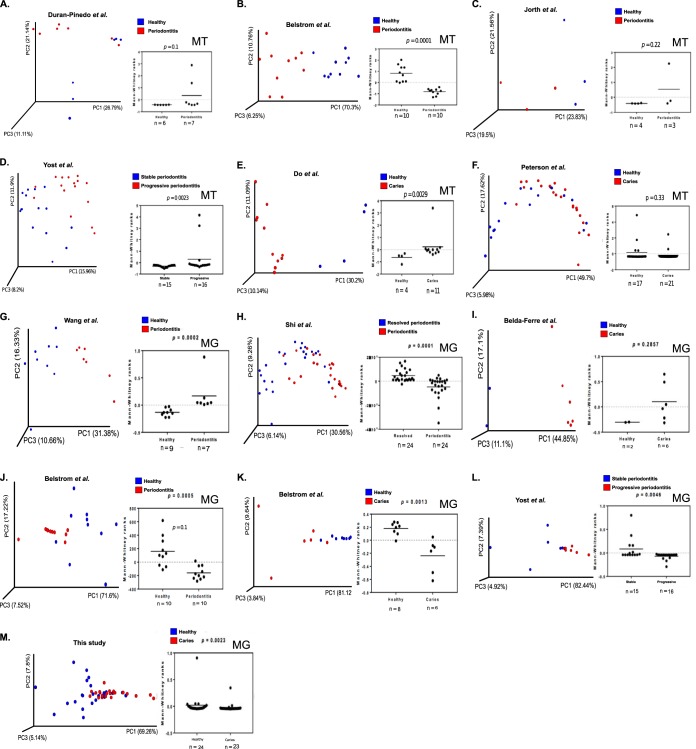
Principal coordinate analysis (PCoA) of BGC profiles representing oral bacterial communities in health and disease. (A) Metatranscriptomics (MT) of BGC profiles from healthy individuals versus individuals with periodontitis from Duran-Pinedo et al. (17). (B) Metatranscriptomics of healthy versus periodontitis BGC profiles from Belstrom et al. (18). (C) Metatranscriptomics of stable versus progressive periodontitis profiles from Jorth et al. (19). (D) Metatranscriptomics of healthy versus caries profiles from Yost et al. (20). (E) Metatranscriptomics of healthy versus caries profiles from Do et al. (21). (F) Metatranscriptomics of healthy versus caries profiles from Peterson et al. (22). (G) Metagenomics (MG) of BGC profiles from healthy individuals versus individuals with periodontitis from Wang et al. (23). (H) Metagenomics of resolved versus periodontitis profiles from Shi et al. (24). (I) Metagenomics of healthy versus caries profiles from Belda-Ferre et al. (25). (J) Metagenomics of healthy versus periodontitis profiles from Belstrom et al. (18). (K) Metagenomics of healthy versus caries profiles from Belstrom et al. (18). (L) Metagenomics of stable versus progressive periodontitis profiles from Yost et al. (20). (M) Metagenomics of healthy versus caries in the current study. PCoA analyses are based on differentially represented BGCs calculated by using the DESeq2 package with false discovery rate (FDR) correction of <0.05. Manhattan distances between samples were visualized using the first three principal coordinates, and significance was tested by applying Mann-Whitney ranks on the most variance captured by the first principal coordinate.

We next systematically examined the differential representation of bacterial BGCs in saliva and dental plaque across 294 human subjects with good oral health, dental caries, or periodontitis. The data from 247 subjects were obtained from eight previous studies, which represented all publicly available metagenomes and metatranscriptomes associated with caries or periodontal disease compared to oral health at the time of this study (28 December 2017; Table S2 posted at https://massive.ucsd.edu/ProteoSAFe/status.jsp?task=1ed182b005a74b94ac730f769b2f37b1). Due to the limited number of publicly available deep sequencing libraries of high quality that represent the same sampling location within the oral cavity (i.e., supra- or subgingival plaque and saliva), we included libraries with mixed sample origin to represent healthy and disease states, respectively (Table S2 posted at https://massive.ucsd.edu/ProteoSAFe/status.jsp?task=1ed182b005a74b94ac730f769b2f37b1). This could have created discrepancies in our analysis, and differences that we present here may represent differences between sampling location and not between oral health states. In addition, DNAs from 47 saliva samples representing 23 children with caries and 24 healthy children were sequenced, and putative BGCs were identified. Nonsupervised exploratory ordination through PCoA revealed significant differences in the representation of BGCs between healthy and diseased subjects in five of the six metatranscriptome studies and six of the seven metagenome studies investigated (Fig. 3). The 1,804 BGCs which were differentially represented in health versus disease in the metagenomes and metatranscriptomes are summarized in Table 1.

**TABLE 1:** Differentially represented or expressed biosynthetic pathways in samples from shotgun metatranscriptomic and metagenomic libraries^*a*^

BGC type	Metatranscriptome (up/down)^*b*^		Metagenome (up/down)^*b*^	
	Caries	Periodontitis	Caries	Periodontitis
Aryl polyene	4/11	29/4	26/5	25/3
Bacteriocin	14/10	1/1	31/1	5/24
Butyrolactone	2/0	1/1	3/0	1/1
Homoserine lactone	0/4	3/4	10/0	1/1
Lantipeptide	7/0	4/0	11/2	1/6
Lassopeptide	0	0	0	0/3
NRPS	7/0	2/8	11/7	10/14
NRPS-PKS hybrid	1/0	0/1	5/0	0/1
Oligosaccharide	66/68	100/40	116/118	287/132
Other	7/0	2/11	36/3	16/15
Phenazine	0	0/1	2/0	0/1
PKS	3/0	1/0	8/0	1/4
Proteusin	0	0	1/0	1/0
Resorcinol	1/4	3/8	4/4	19/0
Sactipeptide	0	0	0	1/0
Siderophore	1/0	1/2	0	1/0
Terpene	1/10	9/1	42/0	1/4
Thiopeptide	1/0	0	0	0/7
Unclassified	93/50	62/121	209/86	300/177

a Number of differentially represented or expressed biosynthetic pathways in saliva, supra- and subgingival plaque samples, from shotgun metatranscriptomic and metagenomic libraries representing 231 subjects (oral healthy individuals [*n* = 110] and individuals with dental caries [*n* = 77] or periodontitis [*n* = 44]). A total of 741 BGCs were differentially abundant in individuals with caries (515 enriched and 226 less abundant), and 1,063 BGCs were associated with periodontitis (670 enriched and 393 less abundant). A total of 355 BGCs were differentially expressed in caries (208 upexpressed and 147 downexpressed), while 421 BGCs were either up- or downexpressed in subjects with periodontitis (218 upexpressed and 203 downexpressed).

b Values in the table are the numbers of BGCs upexpressed/numbers of BGCs downexpressed in individuals with caries or periodontitis.

**FIG 4:**
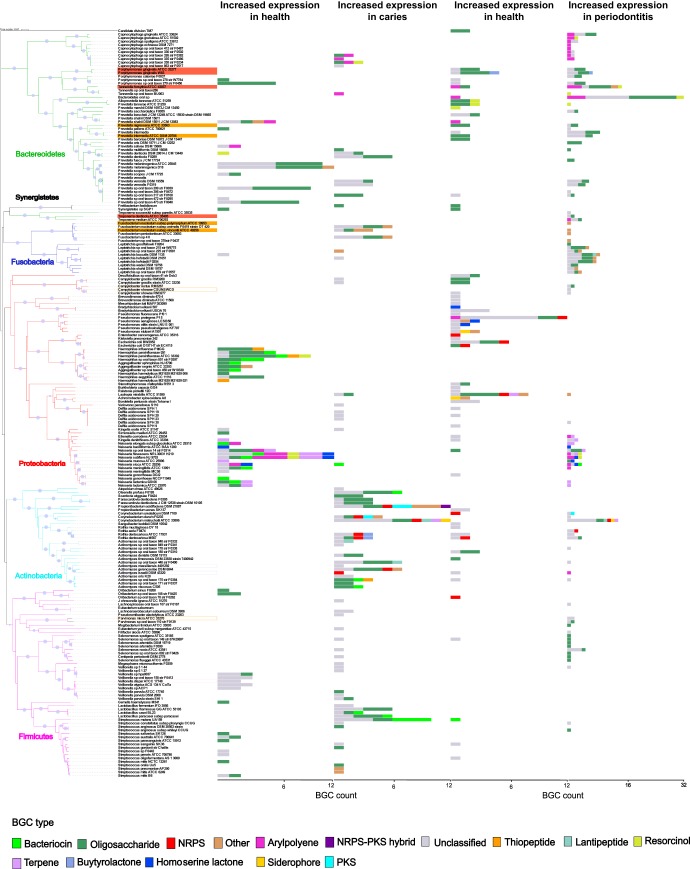
BGCs are differentially expressed in oral health and disease. Bar graphs illustrating phylogenetic distribution of biosynthetic pathways in oral health- and disease-associated oral microorganisms. Taxa with significant changes in BGC expression based on the analyzed metatranscriptomic data sets are shown in the phylogenetic tree on the left. Bacterial genomes associated with periodontal disease and that belong to the red and orange complexes are labeled in orange and red. Bar graphs at the leaf tips display number of BGCs either up- or downexpressed and colored according to the BGC type. It should be noted that the *x*-axis scales are different in the left and right panels. Significant differences in the expression of BGCs were determined based on negative binomial distribution model using DESeq2 with FDR correction (*P* value < 0.05).

The BGCs associated with disease in the metatranscriptome studies were related to the synthesis of a broad range of small molecule types. These types particularly included BGCs of the oligosaccharide, aryl polyene, terpene, bacteriocin, and NRPS classes (Fig. 4). BGCs encoding PKS, NRPS, and bacteriocins from *Actinomyces*, *Rothia*, and *Corynebacterium* had increased expression in subjects with caries, while BGCs encoding terpenes and aryl polyenes from *Neisseria* spp. and *Proteobacteria* had increased expression in healthy subjects (Fig. 4). Previous studies illustrated that aryl polyenes act as protective agents against oxidative stress and that terpenes function as anti-inflammatory agents (40). These findings are in line with previous findings that high levels of *Actinomyces* were associated with severe childhood caries (44). In the caries-associated samples, known cariogenic species belonging to the *Streptococcus, Veillonella*, and *Lactobacillus* genera (45) showed notable changes in bacteriocin and oligosaccharide BGC expression profiles (Fig. 4).

**FIG 5:**
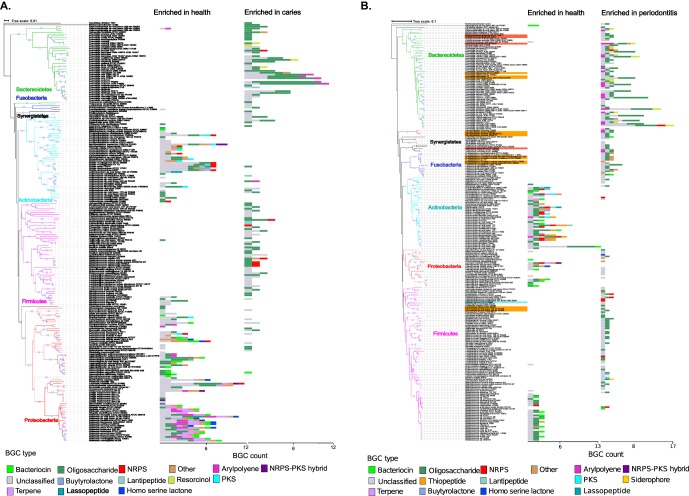
Overview of differentially represented biosynthetic pathways in oral bacterial genomes in oral health and disease states based on metagenomic data sets. Bacterial genomes associated with periodontal disease and that belong to the red and orange complexes are labeled in orange and red. (A) 740 differentially abundant BGCs in the salivary and supragingival microbiome in healthy individuals and individuals with caries, representing 69 subjects (healthy individuals [*n* = 34]; individuals with caries [*n* = 35]). (B) 1,065 differentially abundant BGCs in metagenomes obtained from saliva and subgingival plaque samples from individuals with periodontitis and healthy controls, representing 49 subjects (oral health [*n* = 25]; periodontitis [*n* = 24]). Taxonomic units with significant changes in BGC representation are shown in the phylogenetic tree. Bar graphs at the leaf tips are colored according to the BGC type and display numbers of BGCs expressed higher or lower. Noted that the *x-*axis scales are different for the left and right panels.

In periodontitis, a high number of differentially expressed BGCs (170 BGCs) were identified in community members belonging to the *Bacteroidetes* phylum. Several BGCs encoded by periodontal pathogens of the red and orange complexes (e.g., Porphyromonas gingivalis) were differentially expressed in health compared to periodontal disease. Known red complex species had increased expression of BGCs belonging to the aryl polyene, oligosaccharide, homoserine lactone, and resorcinol classes in diseased states. *Neisseria* spp. also showed interesting signatures, such as increased expression of BGCs belonging to the terpene, resorcinol, bacteriocin, and homoserine lactone classes (Fig. 4). These results suggest that *Neisseria* spp. and its small molecules may play a role in periodontitis, which is worth further explorations in future studies. Homologs to specific BGC products in the MIBiG database which displayed differential expression in health and disease are detailed in Fig. S2. Analysis of the metagenomic studies yielded trends similar to those detailed above (Fig. 5).

10.1128/mBio.00321-19.2 FIG S2 MiBIG annotations for the differentially expressed BGCs in caries and periodontitis. Distance from the center of the graph indicates E values derived from cumulative BLAST bit scores. Concentric demarcations represent close (E value ≥ 1e−290), medium (E value ≥ 1e−180), and distant (E value ≥ 1e−50) homology with reference BGCs. The final circular plot was generated using ggplot2 (26) package in R. Download FIG S2, PDF file, 0.9 MB. Copyright © 2019 Aleti et al. 2019 Aleti et al. This content is distributed under the terms of the Creative Commons Attribution 4.0 International license.

Next, a subset of differentially represented BGCs, which showed high levels of expression in either healthy or diseased states, was examined to determine whether they commonly occur across studies. The results were visualized as a binary occurrence matrix (Fig. S3). In all studies analyzed, only a minor fraction of the differential features (<10 BGCs) were shared between any two studies. The reason for this discrepancy may be due to both biological and technical variations such as differences in anatomical features at the collection site of dental plaque and what time of the day saliva samples were collected. Additionally, sequencing platforms, methodological variations in sample collection, nucleic acid extraction techniques (with or without DNA or RNA/rRNA removal methods), and use of different library preparation methods (see Table S2 posted at https://massive.ucsd.edu/ProteoSAFe/status.jsp?task=1ed182b005a74b94ac730f769b2f37b1 and Fig. S4) may also play important roles. Given the complexity of metagenomes and metatranscriptomes, these factors can certainly influence the sequence composition and lead to the differences we observed here. It is also important to note that due to the fact that BGCs can be horizontally transferred between different bacterial species, mRNA read mapping to BGC databases does not provide definite taxonomic classification and predictions of species-level activities.

10.1128/mBio.00321-19.3 FIG S3 Cooccurrence of differentially represented BGCs across studies. Horizontal bars show numbers of differentially represented BGCs per study, whereas vertical bars indicate the intersection sizes of BGCs. Dark circles connected by vertical lines in the matrix below indicate to which of the studies the shared BGCs belong. BGCs with an overlap size of <10 are not shown. Data from both metagenomic and metatranscriptomic libraries are included in this figure. Download FIG S3, PDF file, 1.8 MB. Copyright © 2019 Aleti et al. 2019 Aleti et al. This content is distributed under the terms of the Creative Commons Attribution 4.0 International license.

10.1128/mBio.00321-19.4 FIG S4 Metagenomic and metatranscriptomic sequence reads analyzed in this study. Box plots showing total numbers of analyzed sequence reads. Individual studies are shown in separate boxes. Metatranscriptomes are shown in the upper panel, and metagenomes are shown in the lower panel. Quality filtered reads that mapped to the reference BGC database after DESeq2 normalization were included in BGC analyses. Sequence reads are presented as log_10_ values. Numbers of reads are provided in Table S1 posted at https://massive.ucsd.edu/ProteoSAFe/status.jsp?task=1ed182b005a74b94ac730f769b2f37b1. Download FIG S4, PDF file, 0.3 MB. Copyright © 2019 Aleti et al. 2019 Aleti et al. This content is distributed under the terms of the Creative Commons Attribution 4.0 International license.

### Differential interactions between BGCs and oral taxa in caries and health.

To examine possible relationships between BGCs and bacterial taxa during health and disease, a focused comparative analysis of the shotgun metagenomics data obtained in this study from saliva samples from healthy children and children with caries (Table S1 posted at https://massive.ucsd.edu/ProteoSAFe/status.jsp?task=1ed182b005a74b94ac730f769b2f37b1) was performed. Children in the caries group had a minimum of two active caries lesions. No overall differences in diet or oral hygiene habits (brushing and flossing) were recorded between the groups. Also, gingiva index scores, which measure oral health were low for both groups (below 1.0) and not significantly different (*P* > 0.05, Table S1 posted at https://massive.ucsd.edu/ProteoSAFe/status.jsp?task=1ed182b005a74b94ac730f769b2f37b1), suggesting that both groups presented with good overall gingival health, except for cavities in the caries active group. Interactions between BGCs and microbial taxa were examined by employing cooccurrence network analysis using the SparCC algorithm, which has the benefit of limiting the number of spurious correlations identified due to species data being compositional (46). While positive correlations were more evident among taxon-taxon relationships (i.e., different taxa benefit from one another’s presence), almost all significant correlations that were identified between specific BGCs and taxa were negative (Table S1 posted at https://massive.ucsd.edu/ProteoSAFe/status.jsp?task=1ed182b005a74b94ac730f769b2f37b1 and Fig. S5 and S6). This suggests that antagonistic relationships, modulated through BGC-produced antimicrobial molecules, are highly significant to the ecology of the oral microbiome.

10.1128/mBio.00321-19.5 FIG S5 Metagenomics-based cooccurrence networks between BGCs and caries-associated microbial community members. Positive correlations are denoted with blue edges, while negative correlations are denoted with red edges. Nodes representing taxa have a white fill, while nodes representing BGCs have a gray fill. (A) Oral microbiomes derived from healthy children. (B) Oral microbiomes derived from children with caries. Download FIG S5, PDF file, 4.9 MB. Copyright © 2019 Aleti et al. 2019 Aleti et al. This content is distributed under the terms of the Creative Commons Attribution 4.0 International license.

10.1128/mBio.00321-19.6 FIG S6 Topological parameters of the cooccurrence networks illustrating correlations between taxa and BGCs in metagenomes derived from healthy children and children with caries. (A) Average numbers of neighbors observed per node are presented as log_10_ values. (B) Numbers of edges (i.e., interactions between neighbors) involved in the network are presented as log_10_ values. (C) Percentage contribution of different BGCs to the total number of correlations in a healthy and caries network. Data from healthy and caries-associated networks are shown in different colors. The average number of neighbors per node and total number of edges were higher in health-associated microbiomes (5,465 interactions among 338 nodes) than in caries-associated microbiomes (3,222 interactions among 274 nodes). The average numbers of neighbors connected per node were 32 and 23.5 in health and caries, respectively, despite the comparable number of nodes. Negative correlations constituted 12% and 4.3% of the total interactions in the health and caries networks, respectively. Download FIG S6, PDF file, 0.8 MB. Copyright © 2019 Aleti et al. 2019 Aleti et al. This content is distributed under the terms of the Creative Commons Attribution 4.0 International license.

All BGCs that had significant correlations to oral taxa (a total of 36) were annotated as close homologs to previously characterized BGCs belonging to the PKS, NRPS, NRPS-PKS hybrid, oligosaccharide, and aryl polyene classes (Fig. S1). In the oral microbiomes derived from healthy children, the interaction network was dominated by negative correlations between oral taxa and BGCs producing glycopeptidolipids, capsular polysaccharides, as well as a homolog of flexirubin (Fig. S5A and Table S1 posted at https://massive.ucsd.edu/ProteoSAFe/status.jsp?task=1ed182b005a74b94ac730f769b2f37b1). The glycopeptidolipids were encoded by the opportunistic pathogens Kytococcus sedentarius and Mycobacterium neoaurum and were primarily shown to vary inversely with the oral taxa *Lactobacillus*, *Prevotella*, *Capnocytophaga*, and *Enterococcus* (Fig. S5A and Table S1 posted at https://massive.ucsd.edu/ProteoSAFe/status.jsp?task=1ed182b005a74b94ac730f769b2f37b1). The flexirubin homolog BGCs were encoded by Actinomyces massiliensis and Prevotella oralis and displayed antagonistic activity against 122 taxa, including Streptococcus mutans, historically considered the primary etiologic species of dental caries (positive correlation coefficients included here range between 0.8 and 1.0, and negative coefficients range between −0.8 and 1) (Fig. S5A and Table S1 posted at https://massive.ucsd.edu/ProteoSAFe/status.jsp?task=1ed182b005a74b94ac730f769b2f37b1). Additional negative correlation with S. mutans ranged between −0.5 and −0.7 (data not shown here). Homologs of the antibiotics bacillaene and pristinamycin (47, 48), harbored by genomes representing Propionibacterium propionicum F0230a and Actinomyces timonensis DSM 23838 displayed negative correlations with several pathogenic taxa: *Lactobacillus*, *Listeria*, *Lysinabacillus*, *Acinetobacter*, *Enterococcus*, *Neisseria*, *Staphylococcus*, *Kingella*, and S. mutans (49) (Fig. S5A). These associations are reminiscent of a previous study which observed similar macrolide-encoding BGCs widely distributed among oral bacterial genomes (2). These macrolide structures were also reported to inhibit the growth of cariogenic streptococci (50). This collective evidence indicates that the isolation and characterization of bacillaene- and pristinamycin-like molecules in future studies may be key to understanding important health-protective mechanisms in the oral cavity. Finally, P. propionicum F0230a encoded a BGC with high sequence homology to a nonribosomal peptide pathway encoding the genotoxin colibactin (51). This BGC showed antagonistic associations with pathogenic genera: *Haemophilus*, *Aggregatibacter*, *Parascardovia*, *Capnocytophaga*, and *Streptococcus* (Table S1 posted at https://massive.ucsd.edu/ProteoSAFe/status.jsp?task=1ed182b005a74b94ac730f769b2f37b1).

Most intriguingly, the number of significant correlations between BGCs and microbial taxa was dramatically reduced in the samples derived from children with caries (Fig. S6A to C). This may indicate that in the oral cavities exhibiting disease, the well-documented colonization resistance of the oral microbiome may be impaired. Of the few significant correlations between BGCs and taxa within the interaction network of the caries-associated microbiome, the vast majority involved BGCs encoding RiPPs with close homology to nosiheptide and hygromycin BGCs (Fig. S5B; Table S1 posted at https://massive.ucsd.edu/ProteoSAFe/status.jsp?task=1ed182b005a74b94ac730f769b2f37b1). The nosiheptide-like BGC, encoded in the genome of Corynebacterium matruchotii, was the most predominant, with antagonistic interactions against ∼90 taxa. These taxa included pathogens from the *Klebsiella*, *Helicobacter*, *Filifactor*, *Haemophilus*, *Enterococcus*, and *Fusobacterium* genera (Fig. S5B; Table S1 posted at https://massive.ucsd.edu/ProteoSAFe/status.jsp?task=1ed182b005a74b94ac730f769b2f37b1). The hygromycin-like BGC from *P. propionicum* negatively correlated with several pathogens belonging to the genera *Lactobacillus*, *Neisseria*, *Klebsiella*, *Anaerococcus*, and *Pseudoramibacter*. Interestingly, there were no significant correlations between S. mutans and BGCs in the caries-associated oral microbiomes, which suggests that during disease, the community lacks the ability to limit the abundance of this cariogenic pathogen. Taken together, these results show that in the oral microbiome, exclusion of particular taxa via antagonistic interactions, mediated by the products of BGCs, is widespread. Although such interactions were still present in the caries-associated oral microbiomes, there were fewer interactions. This underscores the importance of ecology and the role of BGC-produced small molecules in the balance between health and disease.

### Homologs of BGC-produced small molecules are present in oral metabolomes associated with caries and health.

**FIG 6:**
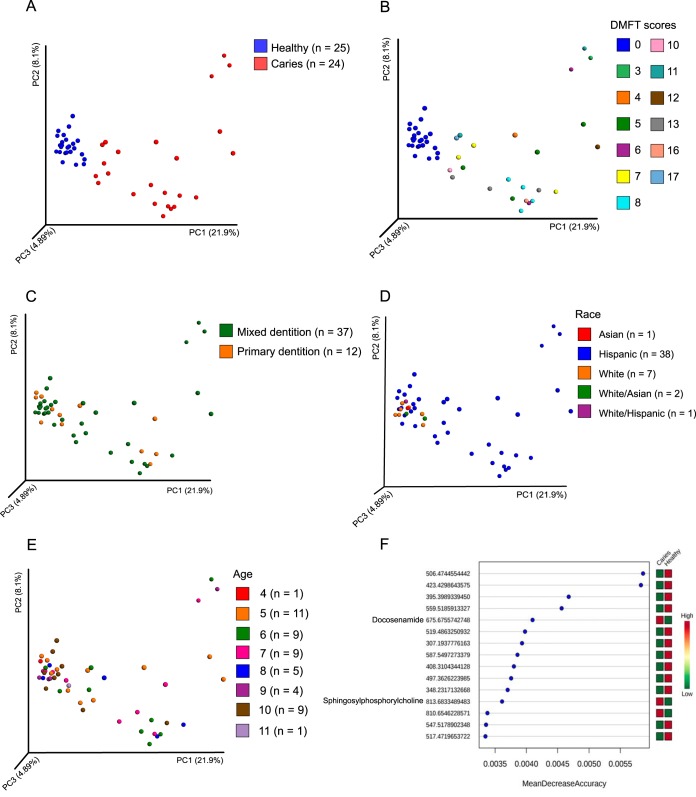
Analysis of small molecules shows clear differences between saliva samples derived from healthy children compared to children with caries. (A) Principal coordinate analysis (PCoA) of parent masses (*m/z*) derived from LC-MS/MS analysis of saliva. Manhattan distances between samples were visualized, and significance was tested by applying Mann-Whitney ranks on the principal coordinates (*P* < 0.0001). (B) PCoA based on DMFT (decayed, missed, or filled teeth) scores (*P* > 0.05). (C) PCoA based on primary and mixed dentition (primary and permanent teeth) (*P* > 0.05). (D) PCoA based on race (*P* > 0.05). (E) PCoA based on age (*P* > 0.05). (F) Fifteen key metabolites responsible for the PCoA separation identified by a random forest importance model.

To validate the production of small molecules from the differentially abundant BGCs that we identified in the analysis above (Fig. 3M), we performed untargeted liquid chromatography-tandem mass spectrometry (LC-MS/MS) analysis of the same saliva samples. Utilizing the Global Natural Products Social Molecular Networking (GNPS) (52) analysis platform, a mass spectral molecular network consisting of 1,369 mass spectral features grouped into 69 molecular families (two or more connected components of a graph) was obtained. Fifty matches were acquired between the query MS/MS spectra and characterized reference spectra from GNPS. To further enhance mass spectrometry annotations and to link annotations to known chemical structures encoded by BGCs, major chemical classes were putatively identified by integrating mass spectral molecular networking with *in silico* annotations and automated chemical classification approaches (53–55). This allowed identification of approximately 38% of the nodes in the mass spectral molecular network at the chemical class level. The most predominant chemical classes within the network were carboxylic acids and derivatives, prenol lipids, fatty acyls, and flavonoids (Fig. S7). Substructures associated with macrolides, terpenoids, and macrolactams were also identified. At the chemical class level, there were no distinct relative abundance patterns between the oral health- and disease-associated samples. This is in stark contrast to PCoA analysis of the 1,369 MS features, which showed clear separation of samples between healthy and diseased states (Fig. 6A) rather than by DMFT scores (Fig. 6B), dentition stage (Fig. 6C), race (Fig. 6D), and age (Fig. 6E), in agreement with the BGC abundance profiles (Fig. 3M). By employing a random forest importance model, 15 key metabolites, which were distinct between healthy and disease states (Fig. 6F), were identified. Of these, 12 were significantly more abundant in healthy subjects, while 3 were more abundant in the subjects with dental caries. Out of the three key metabolites that were significantly more abundant in the diseased subjects, two matches were obtained to lipid compounds from GNPS reference spectra resulting in a level 2 metabolite identification (56). These matches were *N*-nervonoyl-D-erythro-sphingophosphorylcholine and 13-docosenamide. These molecules are likely to originate from the human host and warrant further investigation.

10.1128/mBio.00321-19.7 FIG S7 Differential abundance of chemical features from mass spectrometry data across healthy and diseased subjects. Heatmap illustrating differential abundance of chemical features across healthy and diseased subjects. The heatmap indicates the number of chemical features (nodes within the mass spectral molecular network) per sample and putatively annotated chemical class at the class level. Each column represents a putatively annotated chemical class, and columns are ordered by ClassyFire chemical taxonomy. Rows represent samples from healthy and diseased subjects. The ClassyFire score illustrates the confidence of the putative chemical class identity, where 1 corresponds to a scenario where all putatively annotated structures could be classified within this group and 0 corresponds to a scenario where none of the putatively annotated structures reached a majority consensus chemical class. Download FIG S7, PDF file, 0.2 MB. Copyright © 2019 Aleti et al. 2019 Aleti et al. This content is distributed under the terms of the Creative Commons Attribution 4.0 International license.

Using the *in silico* Network Annotation Propagation tool (NAP) (57), putative structural matches were obtained for 6 out of the 12 key metabolites that were more abundant in the healthy subjects, including terpenoids, phenylpropanoids, as well as fatty alcohols. It should be noted however, that one of the limitations of *in silico* annotation is the uncertainty around the correct structure among the predicted candidate structures. Results should therefore be interpreted with care, and an accurate prediction of the putative identity would require follow-up investigations, which is outside the scope of the present study. It should be also noted that both the genomic and metabolomic approaches employed identify putative homologs and not exact matches. Thus, using current techniques and databases, it is not possible to definitively determine whether the small molecules identified by LC-MS/MS were in fact produced by the specific BGCs predicted by antiSMASH. However, the LC-MS/MS analyses largely support the results of the genomic analyses by detecting classes of small molecules and homologs which were similar to those discovered by the complementary BGC genomics analyses.

### Concluding remarks.

This study significantly expands the number of identified BGCs encoded by bacteria of the human oral microbiome and designates putative products to many novel clusters. Representation and expression of the newly identified BGCs, as well as their relationship to the abundance of oral bacterial taxa were examined during health, dental caries, and periodontitis, revealing significant differences in microbial social ecology and communication among the three host outcomes. This work provides an atlas for further examination and experimental validation of the identified socio-chemical relationships and their role in the pathogenesis of dental caries and periodontal disease. A deeper elucidation of the social activities of the microbes residing in the oral cavity will significantly improve our understanding of the pathogenesis of oral (and extraoral) diseases and will guide development of improved therapeutic strategies to maintain oral health.

## MATERIALS AND METHODS

The ethics statement is provided in Text S1 (Supplementary Materials and Methods section) in the supplemental material.

### Study inclusion/exclusion criteria and collection of saliva.

Subjects were included in the study if the subject was 3 years old or older and in good general health according to a medical history and clinical judgment of the clinical investigator, subjects had at least 12 teeth. Subjects in the caries group had a supragingival (above the gum line) plaque score of 1 or greater on a scale of 0 to 3. Subjects were excluded from the study if they had generalized rampant dental caries, chronic systemic disease, or medical conditions that would influence the ability to participate in the proposed study (i.e., cancer treatment, HIV, rheumatic conditions, history of oral candidiasis). Subjects were also excluded it they had open sores or ulceration in the mouth, radiation therapy to the head and neck region of the body, significantly reduced saliva production or had been treated by anti-inflammatory or antimicrobial therapy. Ethnic origin was mixed for healthy subjects (Hispanic, Asian, Caucasian, Caucasian/Asian), while children with caries were of Hispanic origin. For the latter group, no other ethnic group enrolled despite several attempts to identify interested families/participants. Children with both primary and mixed dentition stages were included (caries group: 18 children with mixed dentition and 6 with primary dentition; healthy group: 19 children with mixed dentition and 6 with primary dentition). To further enable classification of health status (here caries and healthy), a comprehensive oral examination of each subject was performed by a single calibrated pediatric dental resident, using a standard dental mirror, illuminated by artificial light. The visual inspection was aided by tactile inspection with a community periodontal index (CPI) probe when necessary. The number of teeth present was recorded, and dental caries status was recorded using decayed (d), missing due to decay (m), or filled (f) teeth (t) in primary and permanent dentitions (dmft/DMFT), according to the criteria proposed by the World Health Organization in 1997. Duplicate examinations were performed on five randomly selected subjects to assess intraexaminer reliability. Subjects were dichotomized into two groups: caries free (dmft/DMFT = 0) and caries active (subjects with ≥2 active dentin lesions). If the subject qualified for the study, (s)he was asked to brush their teeth after breakfast and abstain from eating and drinking for 2 h prior to saliva collection in the morning. According to a previously developed protocol (58), to ensure that the mouth was free of food or foreign substances, each volunteer rinsed their mouth three times with water 10 min prior to saliva collection. Following the same protocol, we collected approximately 2 ml saliva from each subject by having the subject spit directly in a sterile 15-ml Falcon tube over a 20-min period. Whole saliva was immediately transferred to sterile 2-ml cryovial tubes and snap-frozen for long term storage at −80˚C. For more information, see Text S1 (Supplementary Materials and Methods section) in the supplemental material.

### DNA extraction and metagenomic sequencing.

For detailed protocols of DNA extraction and metagenomic sequencing, see Text S1 (Supplementary Materials and Methods section).

### BGC identification and network analysis of known and putative oral BGCs.

A list of 1,362 described and curated human oral taxa (18 September 2017) was obtained from the Human Oral Microbiome Database (HOMD) (55). A few genomes belonging to transient exogenous bacterial taxa, which also have oral taxon IDs in HOMD were also added to this list (e.g., legume symbiont Mesorhizobium loti, pathogenic Yersinia pestis). In order to identify small-molecule- and secondary metabolite-encoding BGCs in the genomes of bacterial taxa representative of a broad oral bacterial diversity, 461 complete and high-quality draft genomic sequences, annotated as dynamic and static, were obtained from the National Center of Biotechnology Information genome database (https://www.ncbi.nlm.nih.gov/genome), as well as from an in-house database (Table S1 posted at https://massive.ucsd.edu/ProteoSAFe/status.jsp?task=1ed182b005a74b94ac730f769b2f37b1). These sequences were concatenated into a major query-database and fed to antiSMASH (Antibiotics & Secondary Metabolite Analysis Shell, version 4.0) (29). Multiple nucleotide FASTA sequences from BGCs were constructed. We excluded a list of 320 previously described nonbiosynthetic genes commonly found in BGCs (2) (Table S1 posted at https://massive.ucsd.edu/ProteoSAFe/status.jsp?task=1ed182b005a74b94ac730f769b2f37b1) based on text within an attribute using advanced filter settings in CLC Workbench software v. 9. (CLCbio, Aahus, Denmark). The resulting data set contained a total of 192,283 gene sequences from 4,915 BGCs and can be downloaded from the MassIVE repository (ftp://massive.ucsd.edu/MSV000081832) with the accession ID MSV000081832. For more information, see Text S1 (Supplementary Materials and Methods section).

### Comparison of BGCs with known biosynthetic pathways.

A reference MIBiG database comprising multiple amino acid sequences for each BGC was constructed using MultiGeneBlast (30). To further compare BGCs (excluding the fatty acid synthase encoding BGCs) derived from oral bacterial genomes with those encoding the biosynthetic pathways for known compounds, we performed multigene homology searches using complete gene cluster sequences against the MIBiG database by using the stand-alone version of MultiGeneBlast (http://multigeneblast.sourceforge.net/) algorithm with default settings. Subsequently, for each queried BGC, we extracted information from the top hit (with the highest cumulative BLAST bit score) from an output of multiple BLAST hits using an in-house python script. For additional information, see Text S1 (Supplementary Materials and Methods section).

### 16S rRNA gene (16S) phylogenetic analysis.

For a detailed protocol of 16S rRNA gene phylogenetic analysis, see Text S1 (Supplementary Materials and Methods section).

### Metagenomic and metatranscriptomic data collection.

Shotgun metatranscriptomic and metagenomic sequencing data published previously by Duran-Pinedo et al. (22), Belda-Ferre et al. (23), Belstrøm et al. (24), Jorth et al. (59), Do et al. (25), Peterson et al. (26), Yost et al. (27), Wang et al. (28), and Shi et al. (60), as well as our own study of metagenomes from saliva samples obtained from children with good dental health and children with dental caries was analyzed (sequence reads are accessible under BioProject accession no. *PRJNA1234*; Table S2 posted at https://massive.ucsd.edu/ProteoSAFe/status.jsp?task=1ed182b005a74b94ac730f769b2f37b1). For a detailed protocol, see Text S1 (Supplementary Materials and Methods section).

### Differential abundance and expression analyses of BGCs.

We employed a systematic workflow for analyzing abundance and expression profiles of the BGCs. Using SRA toolkit utilities, reads were extracted from metatranscriptome and metagenome shotgun sequenced libraries available via NCBI. For a detailed protocol, see Text S1 (Supplementary Materials and Methods section).

### Principal coordinate analysis.

The differences between samples from healthy versus diseased individuals was investigated by applying principal coordinates analysis (PCoA) on Manhattan distances generated on the DESeq2-normalized count file using the EMPeror (61) tool. For a detailed protocol, see Text S1 (Supplementary Materials and Methods section).

### Correlation network analysis.

The correlation network was constructed using the SparCC algorithm (46) python package (available at https://bitbucket.org/yonatanf/sparcc) to represent both coabundance and coexclusion networks between species and corresponding BGCs. For a detailed protocol, see Text S1 (Supplementary Materials and Methods section).

### Experimental small molecule metabolite detection.

Approximately 150 µl of saliva was lyophilized, and ethyl acetate was added to extract nonpolar molecules. Samples were then vortexed, centrifuged to remove the cell debris, and submitted to untargeted LC-MS/MS analysis. For a detailed protocol, see Text S1 (Supplementary Materials and Methods section).

### Mass spectral molecular networking.

LC-MS/MS spectra obtained from 49 saliva samples (25 healthy subjects and 24 subjects with caries) were preprocessed for feature extraction using MZmine2 (62) and submitted to mass spectral molecular networking through GNPS (43). For a detailed protocol, see Text S1.

### Putative chemical structure annotation.

To putatively annotate chemical structures in our mass spectral molecular networks, we performed *in silico* structure annotation through Network Annotation Propagation (NAP) (57) both for [M+H]+ and [M+Na]+ adducts. For a detailed protocol, see Text S1.

### Availability of data and material.

Sequence data have been submitted to NCBI under BioProject ID PRJNA478018 with SRA accession no. SRP151559. Mass spectral files, LC-MS/MS metadata file, and Nucleotide FASTA sequences of the oral biosynthetic gene cluster collection are accessible from the MassIVE repository (ftp://massive.ucsd.edu/MSV000081832) with the accession ID MSV000081832. Tables S1 and S2 are available at https://massive.ucsd.edu/ProteoSAFe/status.jsp?task=1ed182b005a74b94ac730f769b2f37b1.
