# Supplementary material for: Identification of the Bacterial Biosynthetic Gene Clusters of the Oral Microbiome Illuminates the Unexplored Social Language of Bacteria during Health and Disease
Source: mBio. 2019 Apr 16;10(2):e00321-19. doi: 10.1128/mBio.00321-19 (PMC6469967; doi:10.1128/mBio.00321-19)
Supplement: TEXT S1 [file mBio.00321-19-s0001.docx]

**File S1 - Supplementary Information**

**Supplementary Results**

**Sequence similarity networking reveals unexplored BGC diversity, even in well-studied classes of BGCs**. In the close homology dataset obtained from the MultiGeneBlast analysis, the majority of the oligosaccharide encoding BGCs constituting 41% of the BGCs showed close homology to cell-membrane associated saccharides such as S-layer glycan, O & K antigen capsular lipopolysaccharide, emulsan and diffusible saccharides such as exopolysaccharide, and phosphonate-containing exopolysaccharides (phosphonoglycans) (Fig. S1A and B). Cell-membrane associated saccharides are known to have important roles in microbe-microbe interactions as well as interaction with the host and its immune system for biofilm formation and colonization, while diffusible saccharides provide competitive advantage through antimicrobial activities (1, 2) . We identified BGCs which were widespread among oral Firmicutes, Bacteroidetes, Actinobacteria, Proteobacteria, and Fusobacteria, and displayed significant homology to known aryl polyene-encoding BGCs from *Escherichia coli* and *Vibrio fischeri*. We also found BGCs with close homology to coelibactin, colonic acid, equibactin, glycopeptidolipid, pyoverdine, yersinibactin, aerobactin, and griseobactin which were present in oral Actinobacteria, Proteobacteria and Firmicutes (Fig. 12A and B). Coelibactin is a zinc chelator, while colonic acid is an important form of exopolysaccharide and equibactin is involved in iron acquisition.

**Supplementary Materials and Methods**

**Ethics statement.** Parents and all participants understood the nature of the study and provided informed consent. The Ethics Committees of the School of Dentistry, University of California, Los Angeles, CA, USA and the J. Craig Venter Institute, La Jolla, CA, USA, approved the study design as well as the procedure for obtaining informed consent (reference numbers: 13-001566, 13-001075 and 2016-226). All experiments were performed in accordance with the approved guidelines.

**DNA extraction and metagenomics sequencing**. Frozen saliva samples were thawed on ice and centrifuged at 2,000 x g for 10 minutes to remove eventual eukaryotic cells and solid debris. DNA was extracted and purified from the supernatant by employing QIAmp microbiome (Qiagen) and DNA clean & concentrator (Zymo Research) kit procedures where host nucleic acid depletion step was skipped to maximize bacterial DNA recovery. Purified DNA concentrations ranged between 12 and 14 ng/µL. Libraries were prepared using Illumina NexteraXT DNA library preparation kit according to the manufacturer's instructions. Sequencing was carried out at the J. Craig Venter Institute (JCVI) Joint Technology Center (JTC) by using an Illumina NextSeq 500 platform (San Diego, CA, USA) (150 bp paired end reads). DNA sample concentrations were normalized at prior to sequencing. Barcodes were trimmed and the sequence reads were quality filtered using KneadData pipeline (available at https://bitbucket.org/biobakery/kneaddata) by employing trimmomatic settings of 4-base wide sliding window, with average quality per base >20 and minimum length 90 bp. Similar quality filtering settings were used for all metagenomics and transcriptomics libraries presented in Table S2 posted at https://massive.ucsd.edu/ProteoSAFe/status.jsp?task=1ed182b005a74b94ac730f769b2f37b1. Only sequence libraries that passed quality filtering were included in the analyses.

**BGC identification and network analysis of known and putative oral BGCs.** We identified a total of 5,611 putative and known BGCs representative of oral bacterial genomes. Among these, ~700-fatty acid synthases encoding BGCs, constituting about 13% of the total count were discarded. Furthermore, the format of each cluster was converted from GenBank to FASTA format using the stand-alone FASTA Conversion Tool (Rocap lab <http://rocaplab.ocean.washington.edu/tools/genbank_to_fasta>). antiSMASH often identifies BGCs that encompass multiple gene clusters of different types fused into a single large gene cluster. 63 (~3%) of such unresolved BGCs and were encountered, and were categorized as the ‘complex’ BGC type. For convenience, we combined these BGCs with BGC types ‘Other’ for subsequent analysis. A large number of the putative BGCs (2,665) detected by the ClusterFinder algorithm that did not present clear associations with known BGC types were categorized here as ‘unclassified’.

Mapping of biosynthetic diversity through gene cluster similarity networks was performed as follows. Briefly, Pfam domains from the annotated GenBank files corresponding to the gene clusters defined by antiSMASH and GenBank files from 1,409 experimentally validated gene clusters from MIBIG repository, Minimum Information about Biosynthetic Gene cluster (http://mibig.secondarymetabolites.org) (3) , version 1.3 (September 3rd, 2016) were collectively analyzed using a combination of three weighted indices: Jaccard index, domain sequence similarity score (weighted by sequence identity), and adjacency index with weights 0.36, 0.64 and 0.2 respectively, to define a final distance metric for pairwise comparisons between BGCs based on pfam composition as described previously (4) . Fifteen network files containing all BGC types were generated with different cutoffs ranging from 0.1 to 0.8 with maximum iterations of 1,000. Network files displayed more connections between the clusters with increase in cutoff value.

In order to address the overall diversity and novelty of antiSMASH identified BGCs, a sequence similarity network was generated by calculating pairwise distances between gene sequences derived from major families of BGCs (NRPS, PKS, NRPS-PKS hybrid, aryl polyene, RiPP, oligosaccharide, terpene, and other) using BiG-SCAPE (Biosynthetic Genes Similarity Clustering And Prospecting Engine,<https://git.wageningenur.nl/medema-group/BiG-SCAPE>). A network file with a cutoff value of 0.8 showed good separation of the BGC types and was visualized using Cytoscape version 3.6.0 (http://www.cytoscape.org/index.html) (5).

**Comparison of BGCs with known biosynthetic pathways.** To evaluate the homology of top hits, we computed E-values from the cumulative BLAST bit scores obtained from MultiGeneBlast approach as follows: E = N/2^S^, where N is the size of the search space (N = n*m, where n is the cluster length and m is 43.424502x10^6^, the nucleotide length of the MIBiG database) and S is the cumulative BLAST bit score for each BGC. We then manually assessed the architecture and homology of several gene clusters in order to empirically sort BGCs into close (E < 1 x 10^-290^), medium (E < 1 x 10^-180^), or distant (E < 1 x 10^-50^) homologues. Clusters with E > 1 x 10^-50^ were designated as having no homology to known BGCs in the MIBiG database.

**16S rRNA gene (16S) phylogenetic analysis.** For phylogenetic tree construction, reference 16S sequences were downloaded from the curated HOMD (version 14.51, 3^rd^ January 2017). Multiple-sequence alignment was performed with MUSCLE and a phylogenetic tree was generated by the Neighbor-Joining method using the p-distance model and by implementing a bootstrap test of 1,000 replicates using the Molecular Evolutionary Genetics Analysis (MEGA) software version 7 (6) . antiSMASH-identified BGCs from the genome mining analysis were summarized by taxa and the resulting dataset of BGC count was combined with the phylogenetic tree and visualized using the web-based interactive tree of life (iTOL) version 4 (http://itol.embl.de/) (7).

**Metagenomic and metatranscriptomic data collection.** A total of 294 subjects were analyzed. The metatranscriptome samples analyzed were: healthy control (n = 51), periodontitis (n = 20), stable periodontitis (n = 14), progressive periodontitis (n = 17), and caries (n = 42) (Supplementary Table 3). The metagenome samples analyzed were: healthy control (n = 59), periodontitis (n = 24), stable periodontitis (n = 15), periodontitis progression (n = 16), and caries (n = 35).

**Differential abundance and expression analyses of BGCs.** Sequence reads were aligned against the indexed oral BGC database using Burrows–Wheeler aligner-maximum exact matches algorithm (BWA-MEM) (8) with default parameters. Prior to the alignment step, all known non-biosynthetic genes commonly found in BGCs were excluded from as described in Materials and Methods section. We used a custom Perl script to process the SAM files to extract the number of reads mapped to each BGC in each sample separately. The count file was imported and differential gene expression/abundance between healthy and disease subjects was analyzed by implementing DESeq2 pipeline (9) in R version 3.4.0 (https://www.r-project.org/). DESeq2 employs a geometric normalization strategy for each gene in a dataset. The counts for a gene in each sample is divided by the mean and size factor is then estimated based on the median value of all ratios. This approach corrects the library size and composition bias. Following, DESeq2 employs negative binomial generalized linear models and uses the Wald test for significance testing. In addition, we considered a BGC as active (abundant/expressed) in the datasets of metagenomes and metatranscriptomes if at least half of the core genes within a BGC are detected by a minimum abundance of 10 reads. For significance testing, the adjusted p-values less than a significance level (alpha 0.05) from Wald test was applied as the default inference method. To evaluate whether the under/over represented BGCs occurred across studies, a binary matrix was generated from a total of 2,606 differentially under/over represented BGCs using the presence/absence of BGCs in each study. 1,804 of these BGCs were unique. Datasets with a minimum intersection size of 10 were sorted and visualized in a matrix layout with the UpSetR (10) package. To characterize the taxonomic composition, sequence reads from metagenome shotgun sequenced libraries were also aligned against the in-house oral bacterial genome sequence database (461 bacterial genomes) using BWA-MEM with default settings to generate the taxonomic count table.

**Principal Coordinate analysis.** Only differentially under/over represented BGCs (with FDR correction < 0.05) were considered for the analysis. The amount of variance retained by each Principal Coordinate was further tested by Mann-Whitney ranks test assuming nonparametric distribution of the data by using GraphPad Prism v7. In parallel, we also generated a PCoA plot using the MS1 feature table retrieved from MZmine (see mass spectral molecular networking section) based on Manhattan distances through the in-house tool ClusterApp (available at http://dorresteinappshub.ucsd.edu:3838/clusterMetaboApp0.9.1/).

**Correlation network analysis.** Pairwise correlations were computed separately for caries and healthy control samples based on the raw counts determined for BGCs and taxa, as recommended for SparCC usage (11). Finally, the network files containing both positive and negative correlations were imported and visualized using yFiles layout algorithm in Cytoscape. Topological parameters of the networks were computed using the Cytoscape plugin NetworkAnalyzer (12). Statistical significance of each interaction was then evaluated by setting bootstrap of 100 iterations with two-sided p-values adjusted for multiple comparison correction. To avoid spurious correlations of indirect interactions, we selected correlations with adjusted p-values < 0.05 and with absolute magnitudes ≥ 0.8 (implies a strong co-operative relationship) and ≤ -0.8 (implies a strong antagonistic relationship) using a custom python script. To make biological sense of pairwise interactions, we further filtered out those interactions that occurred exclusively between BGCs. In case of taxa, we considered correlations with a high magnitude of +1 and -1 for visual simplicity.

**Experimental small molecule metabolites detection.** The resuspended extracts were analyzed with an UltiMate 3000 ultrahigh-performance liquid chromatography (UHPLC) system (Thermo Fisher Scientific, Carlsbad, CA) using a Kinetex 1.7-µm C18 reversed-phase UHPLC column (50 by 2.1 mm) and a Maxis qTOF mass spectrometer (Bruker Daltonics, Billerica, MA) equipped with an electrospray ionization (ESI) source. The chromatography was performed at a flow rate of 0.5 ml/min throughout the run. MS spectra were acquired in positive-ion mode in the mass range of m/z 100 to 2,000. An external calibration with ESI-L low-concentration tuning mix (Agilent Technologies, La Jolla, CA) was performed prior to data collection, and internal calibrant hexakis (1H,1H,3H-tetrafluoropropoxy) phosphazene was used throughout the runs. The capillary voltage of 4,500 V, nebulizer gas pressure (nitrogen) of 160 kPa, ion source temperature of 200°C, dry gas flow of 9 liters/min at source temperature, and spectral rate of 3 Hz for MS1 and 10 Hz for MS2 were used. To acquire MS/MS fragmentation, the 10 most intense ions per MS1 were selected. Basic stepping function was used to fragment ions at 50% and 125% of the collision-induced dissociation (CID) calculated for each m/z with a timing of 50% for each step. Similarly, basic stepping of collision radio frequency (RF) of 550 and 800 V peak to peak (Vpp) with a timing of 50% for each step and transfer time stepping of 57 and 90 µs with a timing of 50% for each step was employed. The MS/MS active exclusion parameter was set to 3 and was released after 30 s. The mass of internal calibrant was excluded from the MS2 list.

**Mass spectral molecular networking.** mzXML files were uploaded and batch-processed using MZmine2 (13) with the following settings for each step in the batch: masses were extracted with mass detection module and noise level of 500 counts. Ion chromatograms were built using chromatogram builder with the following parameters: minimum time span of 0.01 min, minimum peak height 1,500 counts, *m/z* and retention time (RT) tolerances at 15 ppm and 0.5 minutes, respectively. Chromatographic peaks were separated and matched to ms2 scans (to create an. mgf file) using the chromatogram deconvolution module with a baseline cutoff algorithm at minimum 1500 counts peak height, peak duration range from 0.01 min to 3.00 min and baseline level of 300. Isotopic chromatograms were combined with the deisotopisation module with *m/z* tolerance of 10 ppm and RT tolerance of 0.1 min. Resulting peaks were aligned with join aligner and *m/z* tolerance of 20 ppm and RT tolerance at 0.1 minutes. Aligned peaks were filtered with peak list raw filter to have at least 3 peaks per row and with minimum of 2 isotopes. An output feature table and .mgf file with detected MS features across all samples and their corresponding abundances were exported for further analysis. The .mgf file was submitted to mass spectral molecular networking through GNPS (14) with parameters described at:

https://gnps.ucsd.edu/ProteoSAFe/status.jsp?task=ac32ab9d21c744ed9eb3879345f10ffa

Two feature tables result from the MZmine preprocessing workflow, a feature table containing all features and a filtered feature table containing only features, for which MS2 spectra were acquired. The unfiltered feature table containing all features was used for PCoA analysis.

**Putative chemical structure annotation.** GNPS spectral library matching resulted in only 50 matches to our experimental MS/MS spectra. To further enhance putative structure annotation, we therefore performed *in silico* annotation through Network Annotation Propagation (NAP) (15). *m/z* tolerance was set to 15 ppm and further parameters are described at:

<https://proteomics2.ucsd.edu/ProteoSAFe/status.jsp?task=dc0578563256468ca9f041e18e8195bd> <https://proteomics2.ucsd.edu/ProteoSAFe/status.jsp?task=a6ab50d6cb5a47af8de708437d94ce64>

Subsequently, we submitted all *in silico,*as well as GNPS library structure, matches to automated chemical classification using ClassyFire (<http://classyfire.wishartlab.com/>) (16) and retrieved consensus classifications per mass spectral molecular family (two or more connected components of a graph) at each hierarchical level of the chemical taxonomy. To visualize differential abundance of chemical features at the chemical class level between diseased and healthy subjects we created a heatmap displaying the number of chemical features (nodes in the mass spectral molecular network) per chemical class and sample. For a chemical feature to be counted, it had to exhibit an intensity of at least 500. A score is calculated in approximation to the confidence of the putative chemical class identity, where 1 corresponds to a scenario, where all putatively annotated structures could be classified within this group and 0, where none of the putatively annotated structures reached a majority consensus chemical class. It should be noted that the consensus chemical classes retrieved are highly dependent upon the structural matches obtained from *in silico* as well as GNPS library matching. Results should therefore be interpreted with care as an accurate putative identification of the chemical structures would require a more thorough investigation, which is out of the scope of the present study. The spectral library matches for N-Nervonoyl-D-erythro-sphingophosphorylcholine and 13-Docosenamide can be accessed at:

<https://gnps.ucsd.edu/ProteoSAFe/result.jsp?task=ac32ab9d21c744ed9eb3879345f10ffa&view=view_all_annotations_DB#%7B%22main.%23Scan%23_lowerinput%22%3A%22766%22%2C%22main.%23Scan%23_upperinput%22%3A%22766%22%7D>

and

<https://gnps.ucsd.edu/ProteoSAFe/result.jsp?task=ac32ab9d21c744ed9eb3879345f10ffa&view=view_all_annotations_DB#%7B%22main.%23Scan%23_lowerinput%22%3A%22213%22%2C%22main.%23Scan%23_upperinput%22%3A%22213%22%7D>.

**REFERENCES**

1. Mazmanian SK, Round JL, Kasper DL. 2008. A microbial symbiosis factor prevents intestinal inflammatory disease. Nature 453:620.

2. Ma C, Chen F, Zhang Y, Sun X, Tong P, Si Y, Zheng S. 2015. Comparison of Oral Microbial Profiles between Children with Severe Early Childhood Caries and Caries-Free Children Using the Human Oral Microbe Identification Microarray. PLOS ONE 10:e0122075.

3. Medema MH, Kottmann R, Yilmaz P, Cummings M, Biggins JB, Blin K, de Bruijn I, Chooi YH, Claesen J, Coates RC, Cruz-Morales P, Duddela S, Düsterhus S, Edwards DJ, Fewer DP, Garg N, Geiger C, Gomez-Escribano JP, Greule A, Hadjithomas M, Haines AS, Helfrich EJN, Hillwig ML, Ishida K, Jones AC, Jones CS, Jungmann K, Kegler C, Kim HU, Kötter P, Krug D, Masschelein J, Melnik AV, Mantovani SM, Monroe EA, Moore M, Moss N, Nützmann H-W, Pan G, Pati A, Petras D, Reen FJ, Rosconi F, Rui Z, Tian Z, Tobias NJ, Tsunematsu Y, Wiemann P, Wyckoff E, Yan X, Yim G, Yu F, Xie Y, Aigle B, Apel AK, Balibar CJ, Balskus EP, Barona-Gómez F, Bechthold A, Bode HB, Borriss R, Brady SF, Brakhage AA, Caffrey P, Cheng Y-Q, Clardy J, Cox RJ, De Mot R, Donadio S, Donia MS, van der Donk WA, Dorrestein PC, Doyle S, Driessen AJM, Ehling-Schulz M, Entian K-D, Fischbach MA, Gerwick L, Gerwick WH, Gross H, Gust B, Hertweck C, Höfte M, Jensen SE, Ju J, Katz L, Kaysser L, Klassen JL, Keller NP, Kormanec J, Kuipers OP, Kuzuyama T, Kyrpides NC, Kwon H-J, Lautru S, Lavigne R, Lee CY, Linquan B, Liu X, Liu W, Luzhetskyy A, Mahmud T, Mast Y, Méndez C, Metsä-Ketelä M, Micklefield J, Mitchell DA, Moore BS, Moreira LM, Müller R, Neilan BA, Nett M, Nielsen J, O’Gara F, Oikawa H, Osbourn A, Osburne MS, Ostash B, Payne SM, Pernodet J-L, Petricek M, Piel J, Ploux O, Raaijmakers JM, Salas JA, Schmitt EK, Scott B, Seipke RF, Shen B, Sherman DH, Sivonen K, Smanski MJ, Sosio M, Stegmann E, Süssmuth RD, Tahlan K, Thomas CM, Tang Y, Truman AW, Viaud M, Walton JD, Walsh CT, Weber T, van Wezel GP, Wilkinson B, Willey JM, Wohlleben W, Wright GD, Ziemert N, Zhang C, Zotchev SB, Breitling R, Takano E, Glöckner FO. 2015. Minimum Information about a Biosynthetic Gene cluster. Nat Chem Biol 11:625–631.

4. Donia MS, Cimermancic P, Schulze CJ, Wieland Brown LC, Martin J, Mitreva M, Clardy J, Linington RG, Fischbach MA. 2014. A Systematic Analysis of Biosynthetic Gene Clusters in the Human Microbiome Reveals a Common Family of Antibiotics. Cell 158:1402–1414.

5. Shannon P, Markiel A, Ozier O, Baliga NS, Wang JT, Ramage D, Amin N, Schwikowski B, Ideker T. 2003. Cytoscape: a software environment for integrated models of biomolecular interaction networks. Genome Res 13:2498–2504.

6. Kumar S, Stecher G, Tamura K. 2016. MEGA7: Molecular Evolutionary Genetics Analysis Version 7.0 for Bigger Datasets. Mol Biol Evol 33:1870–1874.

7. Letunic I, Bork P. 2016. Interactive tree of life (iTOL) v3: an online tool for the display and annotation of phylogenetic and other trees. Nucleic Acids Res 44:W242–W245.

8. Li H. 2013. Aligning sequence reads, clone sequences and assembly contigs with BWA-MEM. arXiv:13033997 [q-bio].

9. Love MI, Huber W, Anders S. 2014. Moderated estimation of fold change and dispersion for RNA-seq data with DESeq2. Genome Biol 15.

10. Lex A, Gehlenborg N, Strobelt H, Vuillemot R, Pfister H. 2014. UpSet: Visualization of Intersecting Sets. IEEE Trans Vis Comput Graph 20:1983–1992.

11. Friedman J, Alm EJ. 2012. Inferring correlation networks from genomic survey data. PLoS Comput Biol 8:e1002687.

12. Assenov Y, Ramírez F, Schelhorn S-E, Lengauer T, Albrecht M. 2008. Computing topological parameters of biological networks. Bioinformatics 24:282–284.

13. Pluskal T, Castillo S, Villar-Briones A, Orešič M. 2010. MZmine 2: Modular framework for processing, visualizing, and analyzing mass spectrometry-based molecular profile data. BMC Bioinformatics 11:395.

14. Wang M, Carver JJ, Phelan VV, Sanchez LM, Garg N, Peng Y, Nguyen DD, Watrous J, Kapono CA, Luzzatto-Knaan T, Porto C, Bouslimani A, Melnik AV, Meehan MJ, Liu W-T, Crüsemann M, Boudreau PD, Esquenazi E, Sandoval-Calderón M, Kersten RD, Pace LA, Quinn RA, Duncan KR, Hsu C-C, Floros DJ, Gavilan RG, Kleigrewe K, Northen T, Dutton RJ, Parrot D, Carlson EE, Aigle B, Michelsen CF, Jelsbak L, Sohlenkamp C, Pevzner P, Edlund A, McLean J, Piel J, Murphy BT, Gerwick L, Liaw C-C, Yang Y-L, Humpf H-U, Maansson M, Keyzers RA, Sims AC, Johnson AR, Sidebottom AM, Sedio BE, Klitgaard A, Larson CB, P CAB, Torres-Mendoza D, Gonzalez DJ, Silva DB, Marques LM, Demarque DP, Pociute E, O’Neill EC, Briand E, Helfrich EJN, Granatosky EA, Glukhov E, Ryffel F, Houson H, Mohimani H, Kharbush JJ, Zeng Y, Vorholt JA, Kurita KL, Charusanti P, McPhail KL, Nielsen KF, Vuong L, Elfeki M, Traxler MF, Engene N, Koyama N, Vining OB, Baric R, Silva RR, Mascuch SJ, Tomasi S, Jenkins S, Macherla V, Hoffman T, Agarwal V, Williams PG, Dai J, Neupane R, Gurr J, Rodríguez AMC, Lamsa A, Zhang C, Dorrestein K, Duggan BM, Almaliti J, Allard P-M, Phapale P, Nothias L-F, Alexandrov T, Litaudon M, Wolfender J-L, Kyle JE, Metz TO, Peryea T, Nguyen D-T, VanLeer D, Shinn P, Jadhav A, Müller R, Waters KM, Shi W, Liu X, Zhang L, Knight R, Jensen PR, Palsson BO, Pogliano K, Linington RG, Gutiérrez M, Lopes NP, Gerwick WH, Moore BS, Dorrestein PC, Bandeira N. 2016. Sharing and community curation of mass spectrometry data with Global Natural Products Social Molecular Networking. Nat Biotechnol 34:828–837.

15. da Silva RR, Wang M, Nothias L-F, van der Hooft JJJ, Caraballo-Rodríguez AM, Fox E, Balunas MJ, Klassen JL, Lopes NP, Dorrestein PC. 2018. Propagating annotations of molecular networks using in silico fragmentation. PLoS Comput Biol 14.

16. Djoumbou Feunang Y, Eisner R, Knox C, Chepelev L, Hastings J, Owen G, Fahy E, Steinbeck C, Subramanian S, Bolton E, Greiner R, Wishart DS. 2016. ClassyFire: automated chemical classification with a comprehensive, computable taxonomy. J Cheminform 8:61.

17. Duran-Pinedo AE, Chen T, Teles R, Starr JR, Wang X, Krishnan K, Frias-Lopez J. 2014. Community-wide transcriptome of the oral microbiome in subjects with and without periodontitis. ISME J 8:1659–1672.

18. Belstrøm D, Constancias F, Liu Y, Yang L, Drautz-Moses DI, Schuster SC, Kohli GS, Jakobsen TH, Holmstrup P, Givskov M. 2017. Metagenomic and metatranscriptomic analysis of saliva reveals disease-associated microbiota in patients with periodontitis and dental caries. NPJ Biofilms Microbiomes 3.

19. Jorth P, Turner KH, Gumus P, Nizam N, Buduneli N, Whiteley M. 2014. Metatranscriptomics of the Human Oral Microbiome during Health and Disease. mBio 5.

20. Yost S, Duran-Pinedo AE, Teles R, Krishnan K, Frias-Lopez J. 2015. Functional signatures of oral dysbiosis during periodontitis progression revealed by microbial metatranscriptome analysis. Genome Medicine 7:27.

21. Do T, Sheehy EC, Mulli T, Hughes F, Beighton D. 2015. Transcriptomic analysis of three Veillonella spp. present in carious dentine and in the saliva of caries-free individuals. Front Cell Infect Microbiol 5.

22. Peterson SN, Meissner T, Su AI, Snesrud E, Ong AC, Schork NJ, Bretz WA. 2014. Functional expression of dental plaque microbiota. Front Cell Infect Microbiol 4.

23. Wang J, Qi J, Zhao H, He S, Zhang Y, Wei S, Zhao F. 2013. Metagenomic sequencing reveals microbiota and its functional potential associated with periodontal disease. Sci Rep 3.

24. Shi B, Chang M, Martin J, Mitreva M, Lux R, Klokkevold P, Sodergren E, Weinstock GM, Haake SK, Li H. 2015. Dynamic Changes in the Subgingival Microbiome and Their Potential for Diagnosis and Prognosis of Periodontitis. mBio 6:e01926-14.

25. Belda-Ferre P, Alcaraz LD, Cabrera-Rubio R, Romero H, Simón-Soro A, Pignatelli M, Mira A. 2012. The oral metagenome in health and disease. ISME J 6:46–56.

26. Wickham H. 2016. ggplot2: Elegant Graphics for Data Analysis. Springer.
