# Supplementary figures and images for: Identification of the Bacterial Biosynthetic Gene Clusters of the Oral Microbiome Illuminates the Unexplored Social Language of Bacteria during Health and Disease
Source: mBio. 2019 Apr 16;10(2):e00321-19. doi: 10.1128/mBio.00321-19 (PMC6469967; doi:10.1128/mBio.00321-19)

Figure S1 A.

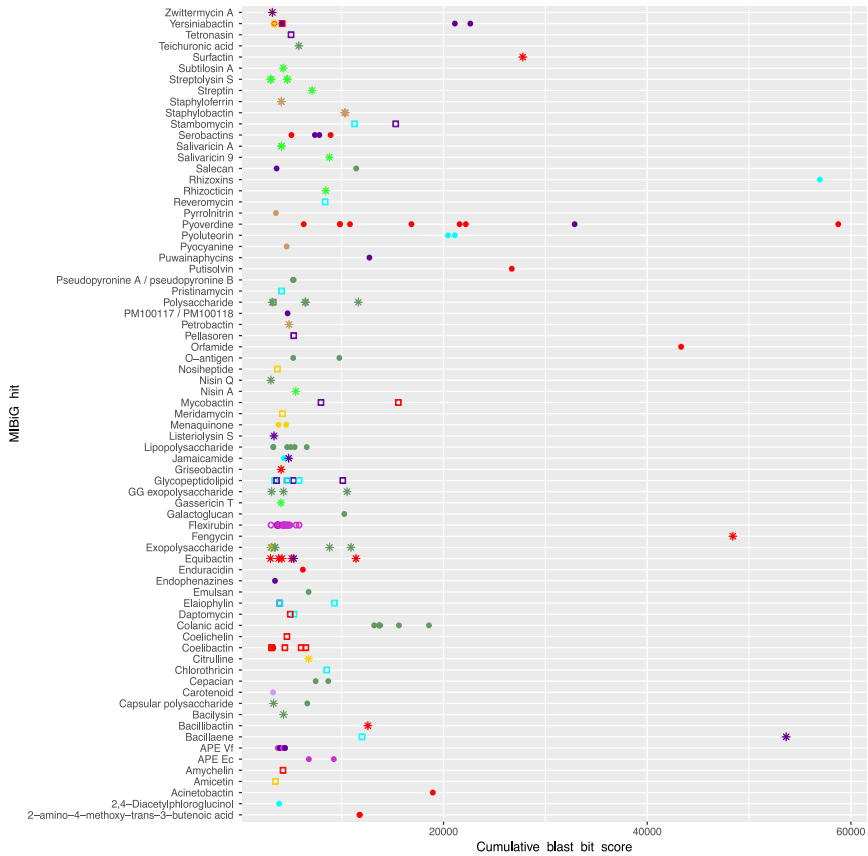

B.

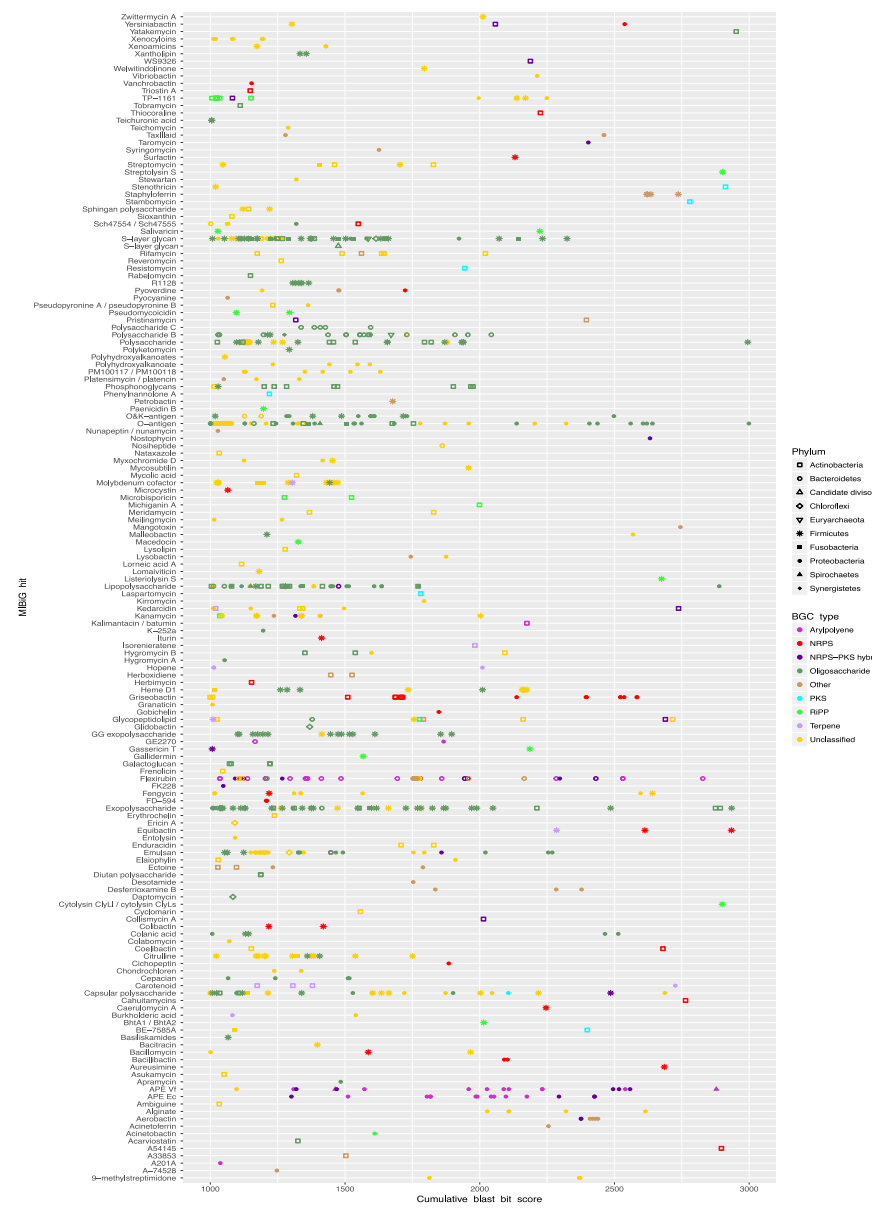

Supplement: FIG S1 [file mBio.00321-19-sf001.pdf]

### Figure S2

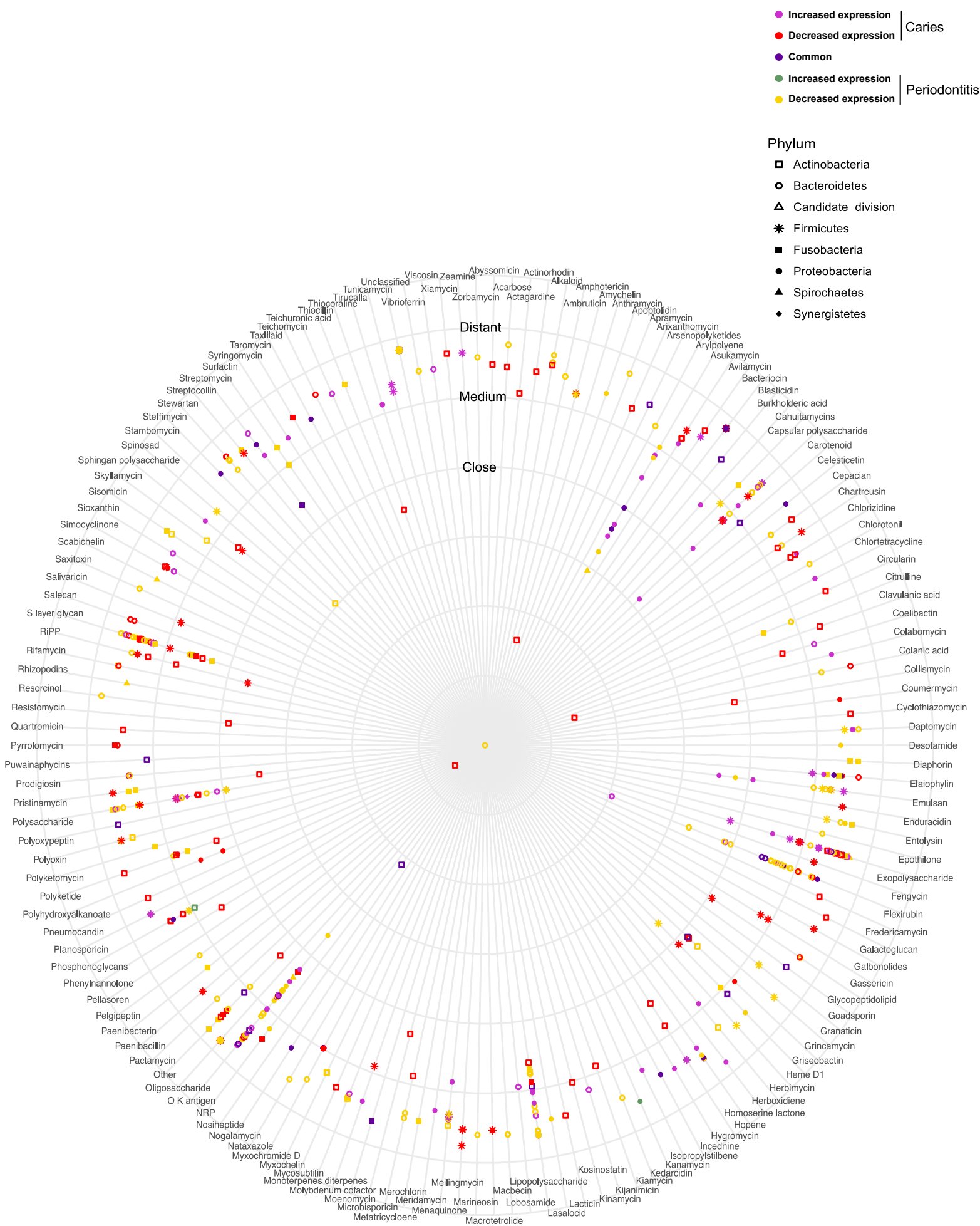

Supplement: FIG S2 [file mBio.00321-19-sf002.pdf]

Figure S3

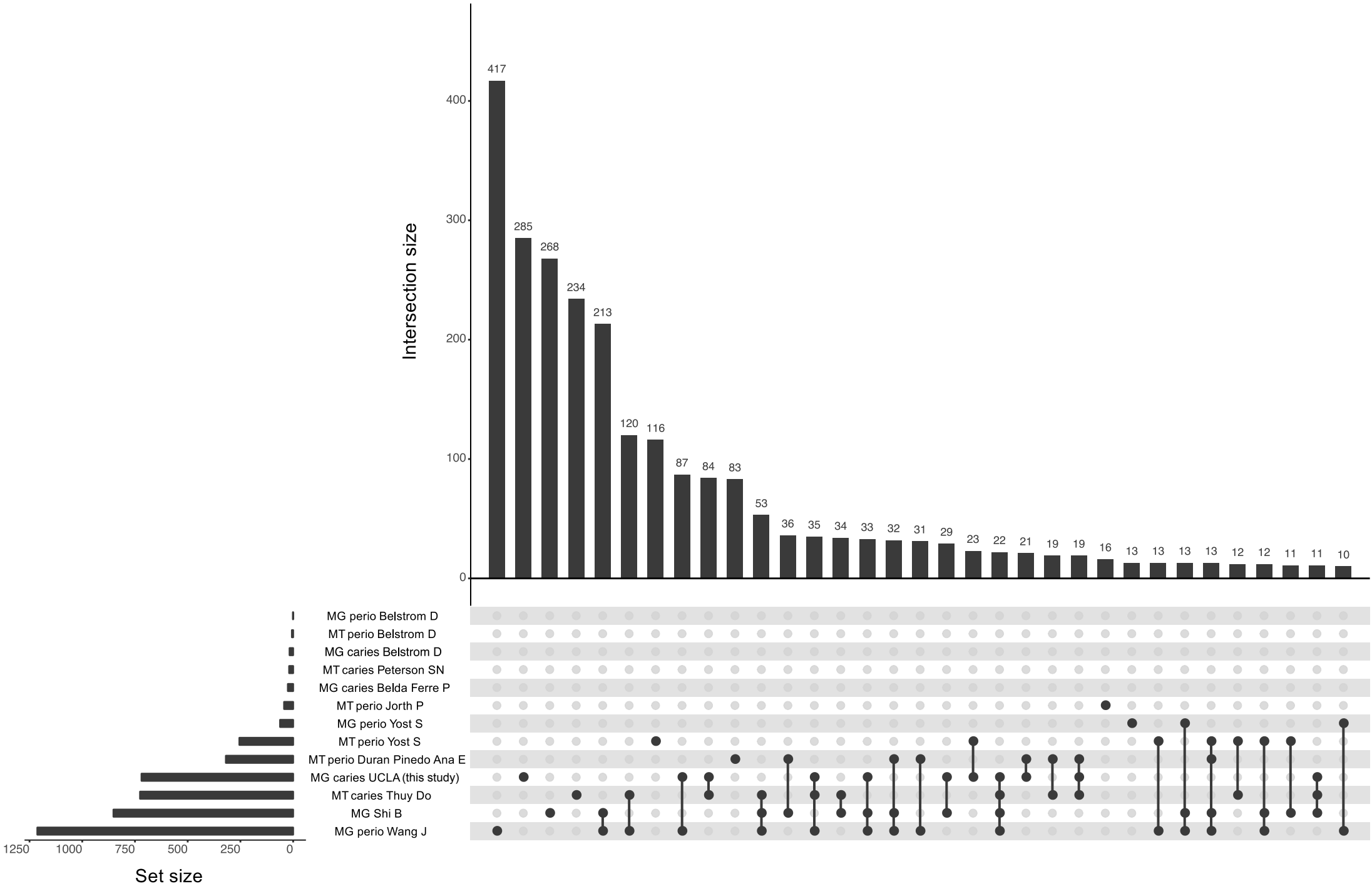

Supplement: FIG S3 [file mBio.00321-19-sf003.pdf]

Figure S4

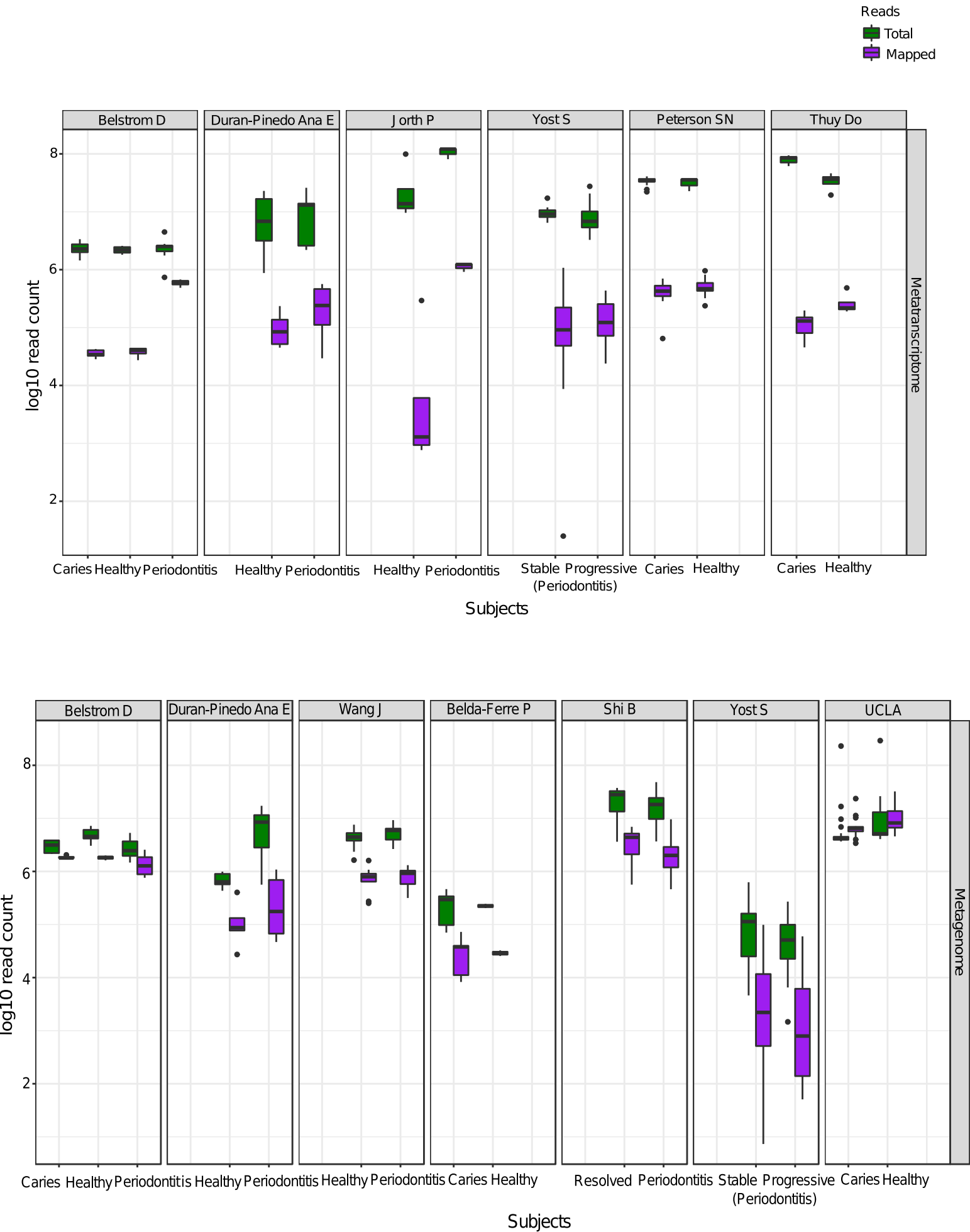

Supplement: FIG S4 [file mBio.00321-19-sf004.pdf]

Figure S5

A.

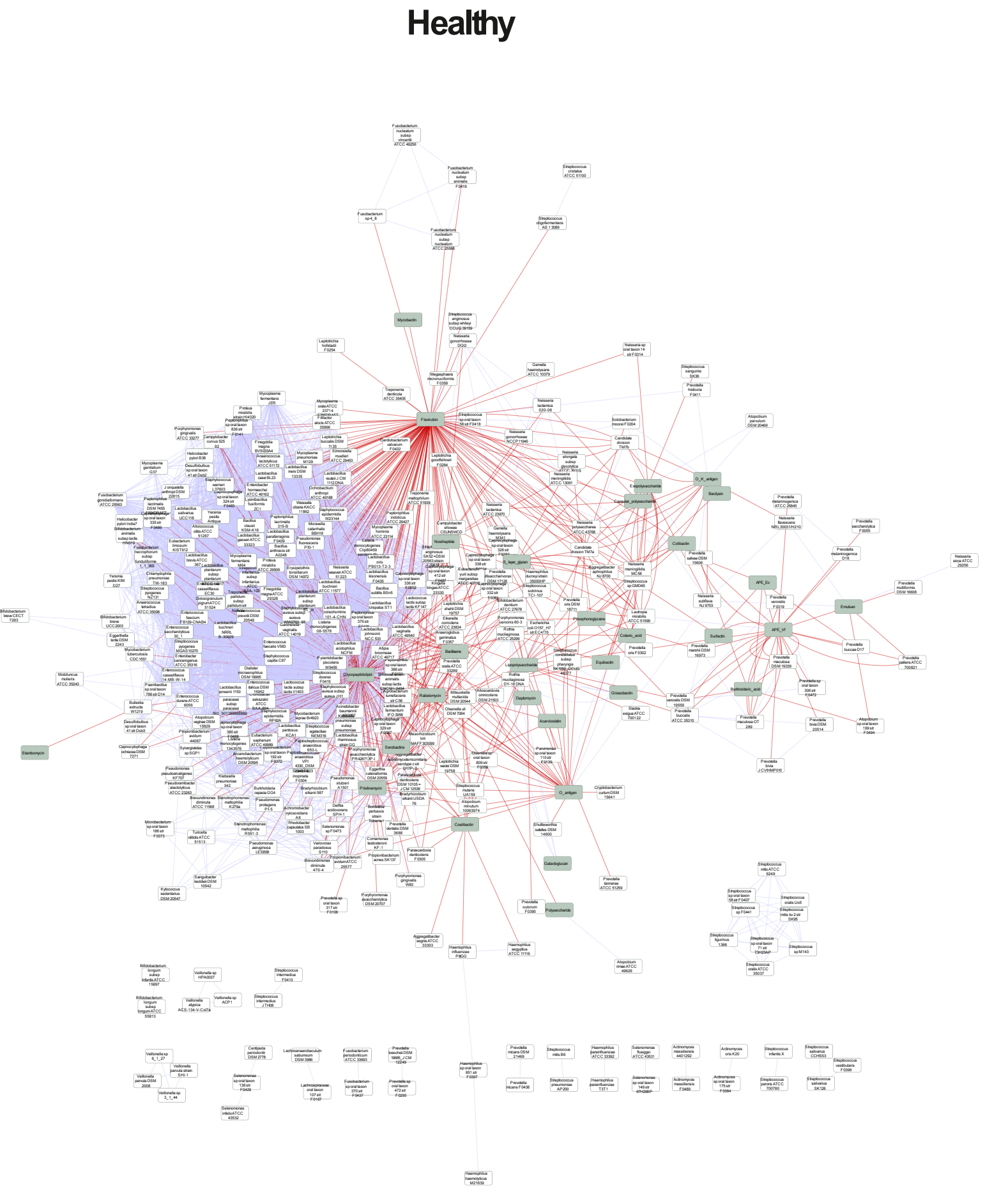

B.

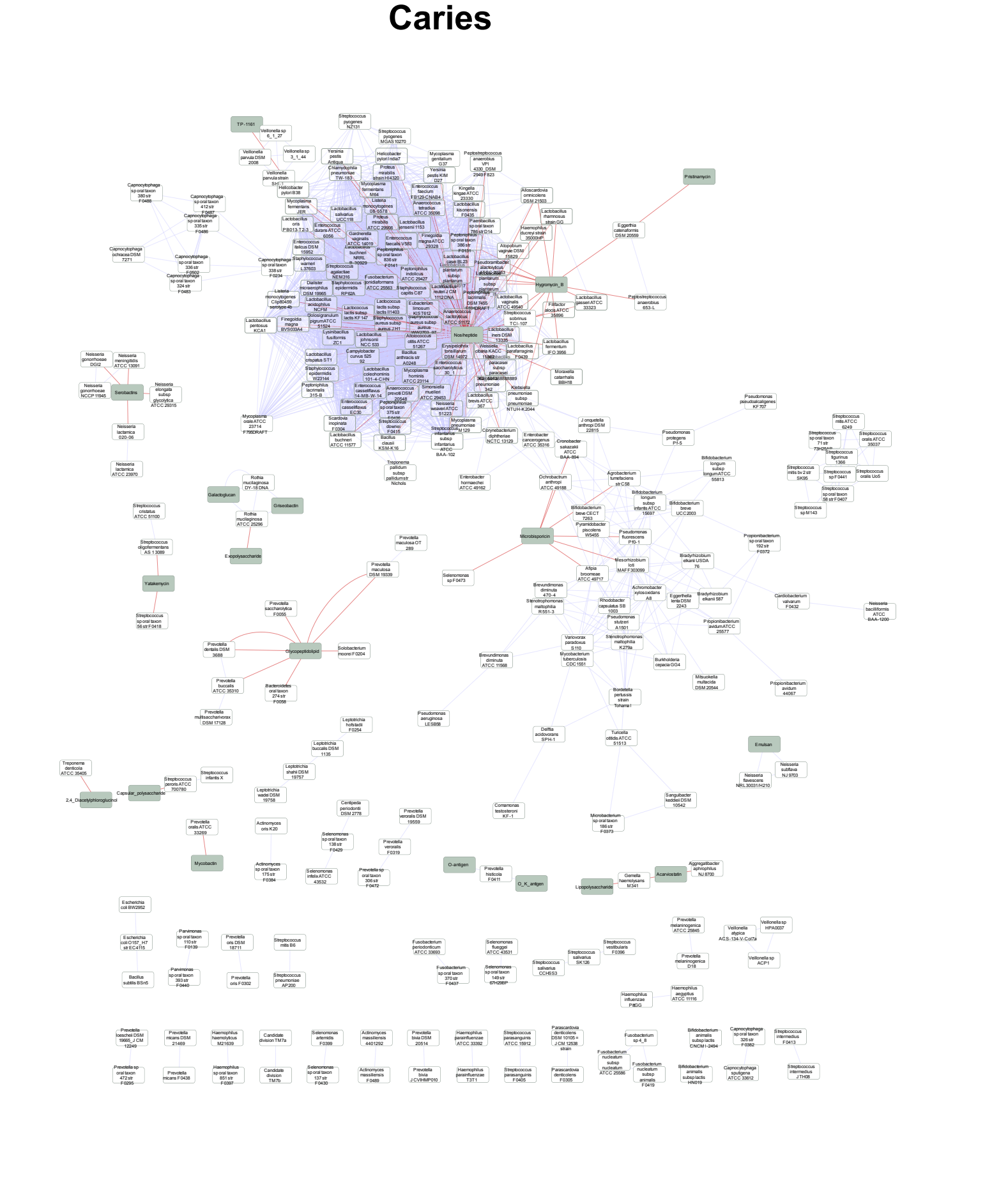

Supplement: FIG S5 [file mBio.00321-19-sf005.pdf]

Figure S6

A.

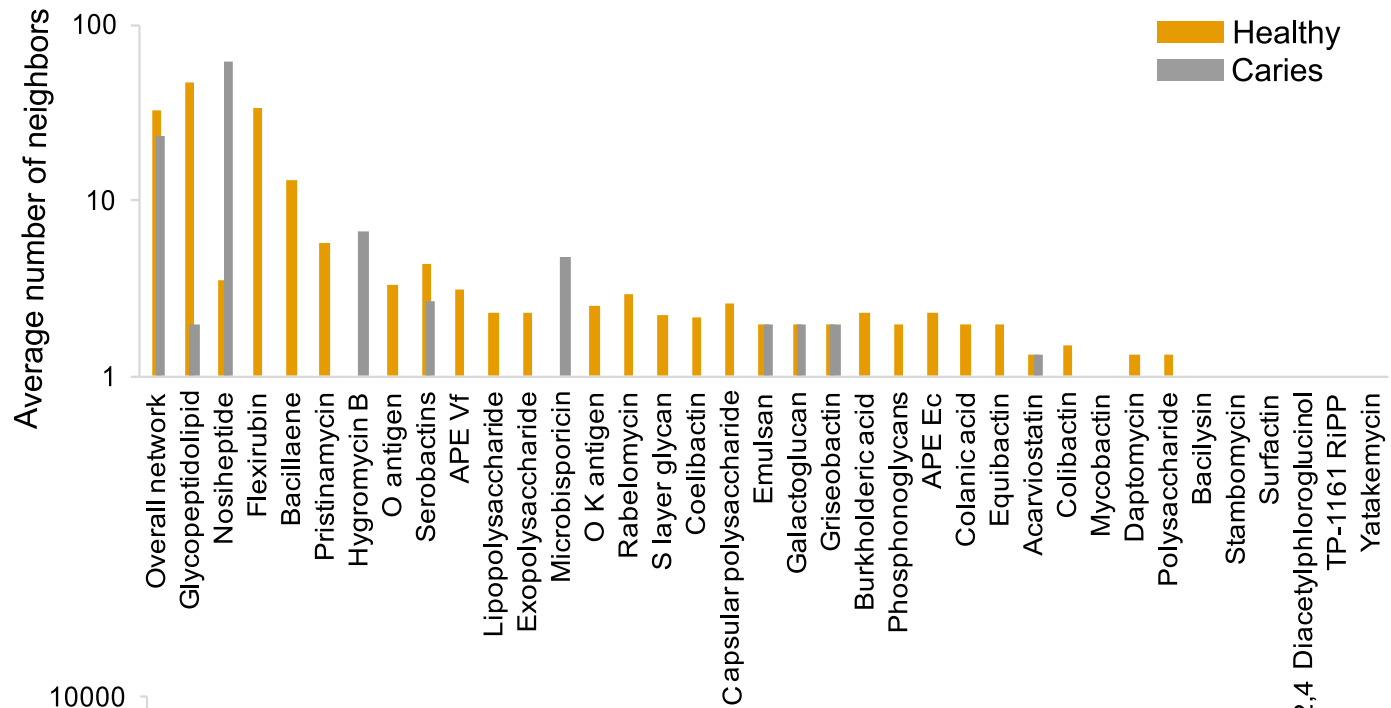

B.

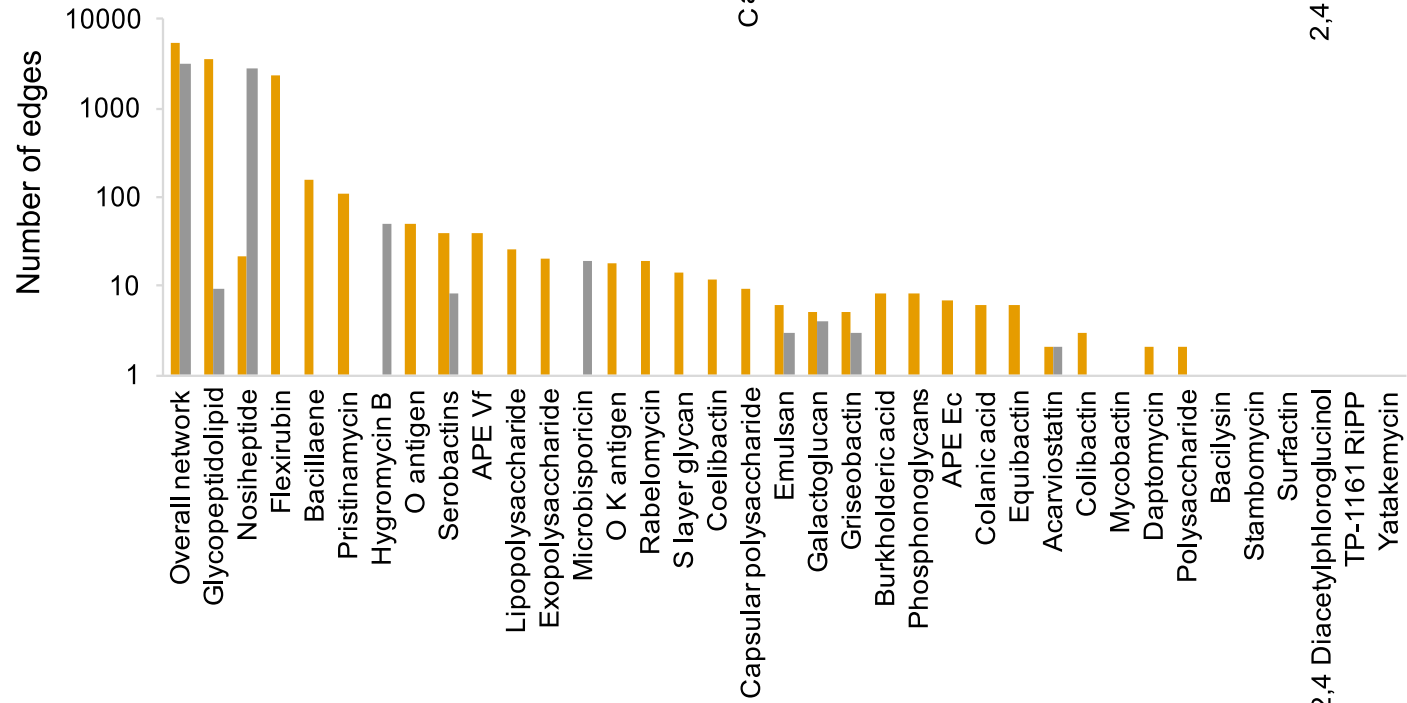

C.

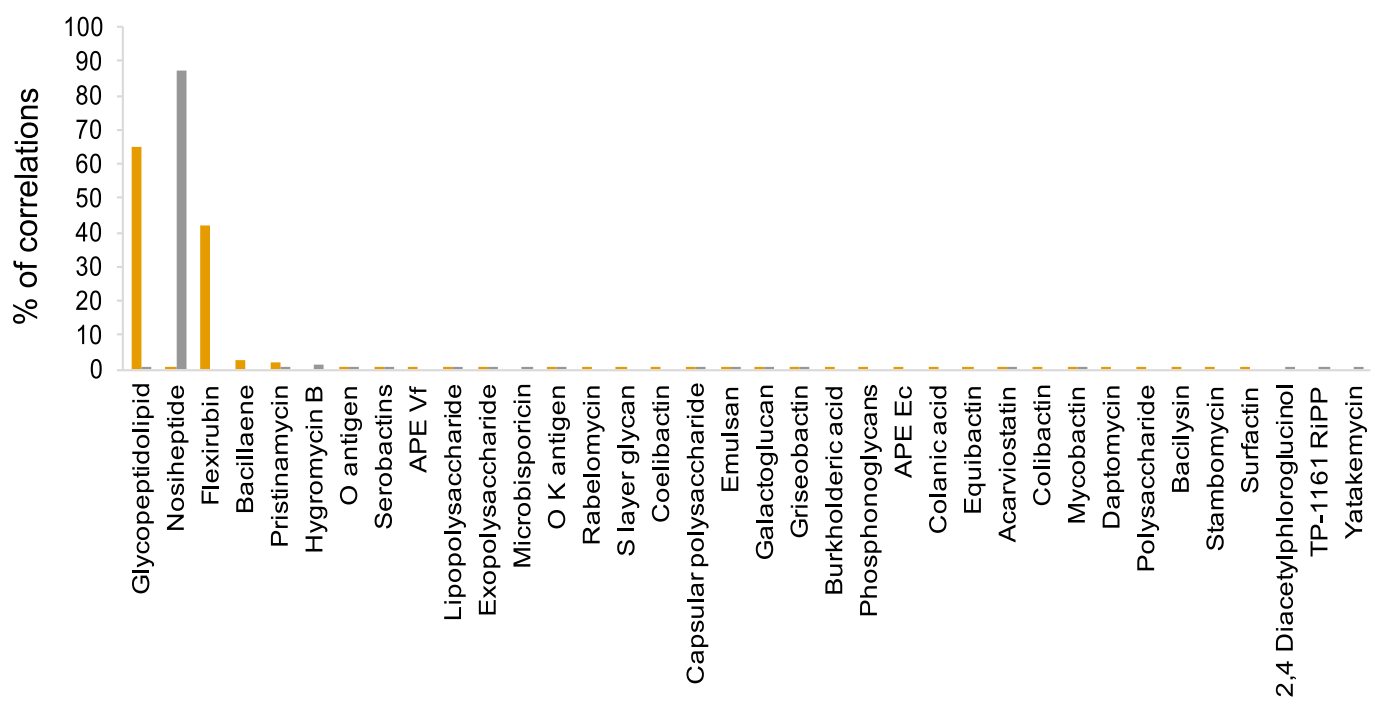

Supplement: FIG S6 [file mBio.00321-19-sf006.pdf]

Figure S7

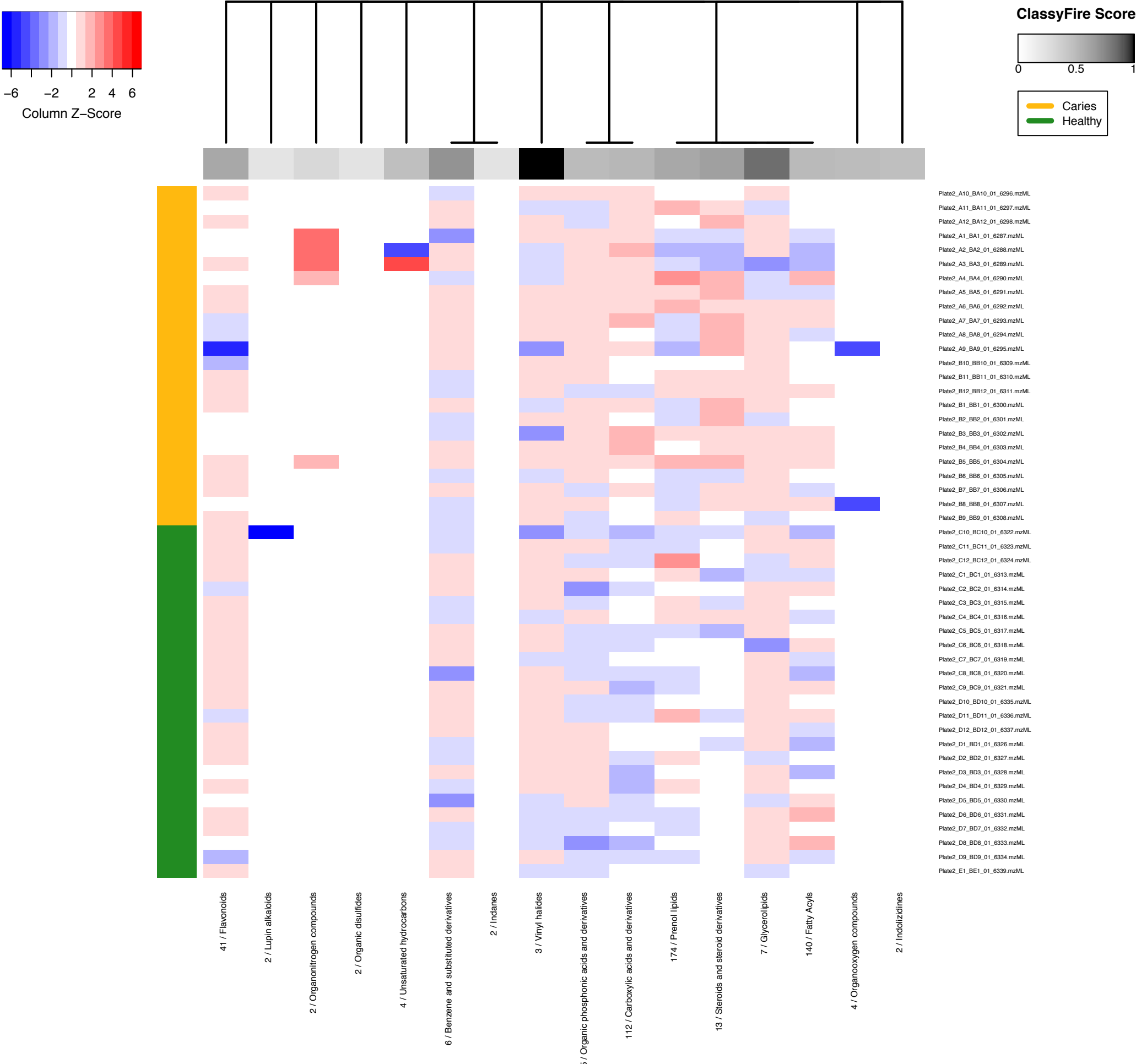

Supplement: FIG S7 [file mBio.00321-19-sf007.pdf]
